# Balancing Edge Defects and Graphitization in a Pt–Fe/Carbon Electrocatalyst for High‐Power‐Density and Durable Flow Seawater‐Al/Acid Hybrid Fuel Cells and Zn–Air Batteries

**DOI:** 10.1002/advs.202308923

**Published:** 2024-09-05

**Authors:** Hao Li, Mengtian Zhang, Mi Wang, Minghao Du, Zijian Wang, Yongxing Zou, Guangxing Pan, Jiaheng Zhang

**Affiliations:** ^1^ Sauvage Laboratory for Smart Materials Harbin Institute of Technology (Shenzhen) Shenzhen 518055 China; ^2^ Research Centre of Printed Flexible Electronics School of Materials Science and Engineering Harbin Institute of Technology Shenzhen 518055 China

**Keywords:** edge‐defects, graphitization, metal–support interaction, Pt‐based catalysts, seawater‐Al/acid hybrid fuel cell, Zn–air battery

## Abstract

Overcoming the trade‐off between the graphitization of the carbon substrate and enhanced electronic metal–support interaction (EMSI) and intrinsic activity of Pt‐C catalysts remains a major challenge for ensuring the durable operation of energy conversion devices. This article presents a hybrid catalyst consisting of PtFe nanoparticles and single Pt and Fe atoms supported on N‐doped carbon (PtFe_NPs_@PtFe_SAs_‐N‐C), which exhibits improved activities in hydrogen evolution and oxygen reduction reactions (HER and ORR, respectively) and has excellent durability owing to the high graphitization, rich edge defects, and porosity of the carbon in PtFe_NPs_@PtFe_SAs_‐N‐C, as well as strong EMSI between the PtFe nanoparticles and edge‐defective carbon embedded with Pt and Fe atoms. According to theoretical calculations, the strong EMSI optimizes the H* adsorption–desorption and facilitates the adsorption OOH*, accelerating the HER and ORR processes. A novel flow seawater‐Al/acid hybrid fuel cell using the PtFe_NPs_@PtFe_SAs_‐N‐C cathode can serve as a high‐efficiency energy conversion device that delivers a high power density of 109.5 mW cm^−2^ while producing H_2_ at a significantly high rate of 271.6 L m^−2^ h^−1^. Moreover, PtFe_NPs_@PtFe_SAs_‐N‐C exhibits a remarkable performance (high power density of 298.0 mW cm^−2^ and long‐term durability of 1000 h) in a flow Zn–air battery.

## Graphical abstract

A carbon‐supported Pt‐based catalyst with balanced defect and graphitization exhibits improved activities and durability in both HER and ORR. And shows excellent performance in a novel flow seawater‐Al/acid hybrid fuel cell and a flow Zn–air battery.

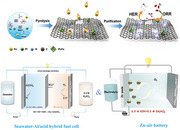

## 1 Introduction

Pt particles dispersed on carbon supports are used as advanced catalysts for the hydrogen evolution reaction (HER) in water‐splitting electrolyzers and the oxygen reduction reaction (ORR) in fuel cells, leading to heavy reliance on Pt as a catalyst source.^[^ ^1^, ^2^, ^3^, ^4^, ^5^, ^6^ ^]^ Challenges in the supply chain and increasing Pt price emphasize the urgent need to develop catalysts with a low Pt loading and enhanced intrinsic performance.^[^ ^7^ ^]^ Reducing the size of Pt particles and anchoring them on carbon support with a high‐surface area are effective methods for increasing the utilization of Pt and improving its catalytic activity.^[^ ^8^, ^9^, ^10^, ^11^ ^]^ In addition to regulating geometric configurations of platinum particles, the carbon support also plays a key role in the overall performance of the composite catalyst. An excellent carbon support has a high Brunauer–Emmett–Teller (BET) surface area, high conductivity, balanced graphitization and defects, and sufficient porosity, which are favorable for not only dispersing the Pt particles but also facilitating electron/mass transfer and durability via metal–support interactions. However, the practical application of the prevailing Pt‐C‐based catalysts is hindered by their poor durability owing to carbon corrosion and weak electronic metal–support interaction (EMSI),^[^ ^12^, ^13^, ^14^, ^15^ ^]^ resulting in Pt particle dissolution, detachment, agglomeration, and Ostwald ripening.

To rationally design carbon supports with desired structural and chemical properties, defect generation and increasing the graphitization of the carbon support are vital, as they increase the accessible internal surface area, conductivity, and enrich coordination environment, thus significantly improving the electrocatalytic performance of the supported metal particles.^[^ ^16^, ^17^, ^18^, ^19^ ^]^ A carbon support rich in defects can provide an unsaturated coordination environment for trapping metal atoms via the formation of thermodynamically favorable metal–support bonds. This facilitates the generation of stable sub‐nanometer metal particles with well‐defined active centers or even single metal atoms, resulting in a remarkable size effect in catalytic reactions.^[^ ^20^, ^21^, ^22^, ^23^, ^24^, ^25^ ^]^ Moreover, a strong EMSI can modulate the *d*‐band center of the atomic metal site, facilitating the adsorption and desorption of reaction intermediates, which lowers the energy barrier and facilitates the rate‐limiting reaction.^[^ ^8^, ^18^, ^26^, ^27^ ^]^

The doping of non‐metallic heteroatoms, such as N, P, S, O, and Se, into the carbon skeleton is a common method for introducing extrinsic defects. Owing to their difference in electronegativity or size with respect to C, these heteroatoms cause charge‐density and/or spin‐density redistribution and modulate the free energy for the adsorption of key intermediates.^[^ ^28^, ^29^ ^]^ Moreover, the introduction of metal into the carbon support, As a new extrinsic defect type, can further induce charge redistribution or modify the electron density state of the active metal sites through electron transfer, thereby creating a more diverse and intricate electronic environment within the carbon support.^[^ ^25^ ^]^ And the incorporation of metal atoms into the carbon skeleton can diversify carbon defects and reconstruct the carbon structure. For example, transition metals (Fe, Co, and Ni)^[^ ^26^, ^30^ ^]^ and alkaline‐earth metals (K, Na, and Mg)^[^ ^31^, ^32^ ^]^ can etch the carbon skeleton to create pores and edge defects. Unfortunately, the carbon materials rich in extrinsic defects often exhibit amorphous characteristics with a low degree of graphitization. Consequently, they are electrochemically unstable owing to severe carbon corrosion during catalytic reactions, especially under harsh conditions, such as a high temperature, low pH, or high oxygen content. Many studies on carbon supports have extensively focused on extrinsic defect engineering, the significance of intrinsic defects, including edges, vacancies, holes, or topological defects,^[^ ^8^, ^9^, ^27^, ^33^ ^]^ is often underestimated. In addition, according to the second law of thermodynamics, intrinsic carbon defects exist in all forms of carbon materials, even in highly graphitized graphene, where edge defects persist.^[^ ^34^ ^]^ Therefore, the rational construction of intrinsic defects while maintaining a graphitic carbon substrate is a feasible approach to overcome the trade‐off between the graphitization of carbon substrates and enhanced EMSI and intrinsic activity of Pt‐C‐based catalysts. However, this approach has rarely been explored, mainly because of the frequently overlooked intrinsic defects, and the chemical inertness of graphitized carbon.

Herein, we report a highly durable and active Pt‐based catalyst with PtFe nanoparticles and edge‐defective N‐doped carbon embedded with Pt and Fe dual atoms (denoted as PtFe_NPs_@PtFe_SAs_‐N‐C). We introduced a Fe precursor during the carbothermal reduction process to take advantage of metal‐catalyzed graphitization for enhancing the degree of graphitization and durability of the carbon substrate.^[^ ^35^, ^36^, ^37^ ^]^ On the other hand, Fe species etch the carbon skeleton to create edge defects and pores, which can trigger the redistribution of electrons between adjacent single Pt and Fe atoms, PtFe nanoparticles, and defective carbon substrates, resulting in a strong EMSI, which is expected to significantly improve the activity and stability of the PtFe_NPs_@PtFe_SAs_‐N‐C catalyst. Indeed, the PtFe_NPs_@PtFe_SAs_‐N‐C demonstrated superior catalytic activity and stability in the HER and ORR, as confirmed by the low overpotential of 41 mV in the HER and half‐wave potential of 0.895 V in the ORR. In addition, PtFe_NPs_@PtFe_SAs_‐N‐C exhibits remarkable stability of up to 270 h in the HER and 220 h in the ORR. More interestingly, the exceptional HER activity of the PtFe_NPs_@PtFe_SAs_‐N‐C catalyst enabled the development of a novel flow seawater‐Al/acid hybrid fuel cell (FSAAH), which functions as a self‐powered system for continuous hydrogen generation at a significantly high rate of 271.6 L m^−2^ h^−1^ and a high‐power‐density of 109.5 mW cm^−2^. In addition, a flow Zn–air battery (ZAB) assembled using the PtFe_NPs_@PtFe_SAs_‐N‐C catalyst as the cathode could deliver a high power density of 298.0 mW cm^−2^ and outstanding cycle stability of 1000 h.

## 2 Results and Discussion

### 2.1 Synthesis and Characterization

**Figure 1:**
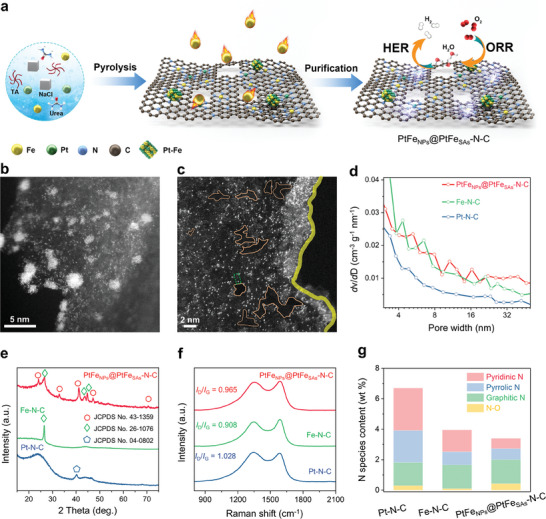
Morphological and structural characterization of the catalysts. a) Schematic illustration of the preparation of PtFe_NPs_@PtFe_SAs_‐N‐C. b,c) Aberration‐corrected HAADF‐STEM images of PtFe_NPs_@PtFe_SAs_‐N‐C (the nanopores are marked with brown irregular polygons, and the Pt and Fe atoms are marked with green dashed rectangles. The intensity profiles of Fe/Pt atoms are displayed in Figure S1, Supporting Information). d) Pore‐size distributions of PtFe_NPs_@PtFe_SAs_‐N‐C, Fe‐N‐C, and Pt‐N‐C. e) XRD patterns of PtFe_NPs_@PtFe_SAs_‐N‐C, Fe‐N‐C, and Pt‐N‐C. f) Raman spectra of PtFe_NPs_@PtFe_SAs_‐N‐C, Fe‐N‐C, and Pt‐N‐C. g) N species distribution in PtFe_NPs_@PtFe_SAs_‐N‐C, Fe‐N‐C, and Pt‐N‐C.

The procedure for synthesizing the catalyst with Pt‐Fe active sites anchored on the edge‐defective carbon structure is illustrated in **Figure** **1**a. First, a precursor solution was prepared for spray drying, in which tannic acid was used as the chelating agent because it strongly chelates to metal ions, urea was used as the nitrogen source, NaCl served as the template, and K_2_PtCl_4_ and FeCl_2_ were employed as the metal sources. The mixed solution was spray‐dried and then activated by air oxidation. During high‐temperature treatment, the molten NaCl shaped the carbon structure into ultrathin nanosheets.^[^ ^38^, ^39^, ^40^ ^]^ More importantly, the Fe source etched the carbon skeleton, creating additional pores and abundant edge defects^[^ ^26^ ^]^ while the Fe‐catalyzed transformation of the carbon skeleton into ordered graphitized carbon occurred.^[^ ^36^ ^]^ The so‐formed edge‐defective carbon support could preferentially anchor single metal atoms at the edge sites. Finally, a purifying acid treatment was conducted to remove unstable metal species, and the PtFe_NPs_@PtFe_SAs_‐N‐C electrocatalyst was obtained. To facilitate comparison, single‐metal Fe‐N‐C and Pt‐N‐C catalysts were prepared using an identical synthetic procedure, without adding FeCl_2_ or K_2_PtCl_4_.

The morphological and structural features of PtFe_NPs_@PtFe_SAs_‐N‐C were examined using scanning electron microscopy (SEM) and transmission electron microscopy (TEM) (Figure S1a–c, Supporting Information). Under the assistance of molten NaCl, PtFe_NPs_@PtFe_SAs_‐N‐C formed hollow, thin‐walled spheres and carbon nanosheets, and no large metal particles were observed in the TEM images. In Figure S1d,e (Supporting Information), the high‐resolution TEM (HRTEM) images reveal the nanoparticles with a 0.22 nm lattice spacing, consistent with the (220) plane of the PtFe alloy. In addition, numerous pores were scattered on the carbon support. In Figure S1f–j (Supporting Information), the energy‐dispersive X‐ray spectrometry (EDS) results reveal that elemental Fe and Pt were well distributed over the entire carbon support, indicating the presence of Fe and Pt in single‐atom states in addition to the nanoparticle. Aberration‐corrected high‐angle annular dark‐field scanning TEM (HAADF‐STEM) was used for a more detailed visualization of the dispersion of the Pt and Fe single atoms. Figure 1b shows that the nanoparticles were surrounded by abundant isolated bright and dark dots. As Pt atoms have a higher *Z*‐contrast than Fe atoms (Z_Fe_(26) < Z_Pt_(78)), these bright and dark dots can be identified as single Pt and Fe atoms, respectively (Figure 1c), as further evidenced by their line scan intensity profiles (Figure S2, Supporting Information). These results clearly demonstrate the coexistence of abundant single atoms of Pt and Fe and PtFe nanoparticles in PtFe_NPs_@PtFe_SAs_‐N‐C. Interestingly, the single metal atoms were concentrated at the edge areas, in addition to the basal planes, as marked by the brown circle in Figure 1c, leading to an electron‐enriched edge. The electron‐enriched edge can influence the electronic states of the Pt 5*d* and Fe 3*d* orbitals, thus altering the catalytic activities of Pt and Fe.^[^ ^41^, ^42^ ^]^ In Table S1 (Supporting Information), the mass loadings of Pt and Fe in PtFe_NPs_@PtFe_SAs_‐N‐C were quantified via inductively coupled plasma optical emission spectrometry (ICP‐OES, 2.2 wt.% for Pt and 0.84 wt.% for Fe).

The specific surface areas and pore structures of the catalysts were assessed by Nitrogen adsorption–desorption isotherms (Figure S3, Supporting Information). All samples provided Type IV isotherms, suggesting the existence of a hierarchical structure with micropores and mesopores, which can facilitate mass transport during the catalytic reaction.^[^ ^43^ ^]^ Notably, the Fe precursor (FeCl_2_) had a positive effect on increasing the surface area of the carbon substrate, as confirmed by the significantly higher specific surface area (1028.82 m^2^ g^−1^) of the PtFe_NPs_@PtFe_SAs_‐N‐C catalyst compared with those of Pt‐N‐C (712.95 m^2^ g^−1^) and Fe‐N‐C (876.37 m^2^ g^−1^). By adjusting the quantity of FeCl_2_, a set of Fe‐*x*‐Pt‐N‐C catalysts were obtained (*x* refers to the amount of FeCl_2_; *x* = 100, 300, and 500 mg; Fe‐300‐Pt‐N‐C is PtFe_NPs_@PtFe_SAs_‐N‐C). The specific surface area of the Fe‐*x*‐Pt‐N‐C catalyst initially increased and then decreased with the increase in the Fe content (Fe‐100‐Pt‐N‐C: 820.94 m^2^ g^−1^, Fe‐300‐Pt‐N‐C: 1028.82 m^2^ g^−1^, and Fe‐500‐Pt‐N‐C: 762.95 m^2^ g^−1^). Fe‐500‐Pt‐N‐C had a lower specific surface area than Fe‐300‐Pt‐N‐C, This was due to the excessive addition of FeCl_2_, which induced the partial collapse of the pore structure.^[^ ^26^ ^]^ The pore‐size distributions in Figure 1d and Figure S3f (Supporting Information) reveal that the addition of FeCl_2_ can generate new mesopores in the carbon structure, leading to increased mesopore volume compared to the Fe‐free catalyst (Pt‐N‐C). The generation of numerous mesopores creates more edge defects, which can modulate the local density of the π‐electrons and increase the chemical reactivity. Thus, an optimal amount of Fe can adequately etch the catalyst surface, leading to a larger specific surface area and more edge defects, as observed in PtFe_NPs_@PtFe_SAs_‐N‐C.

X‐ray diffraction (XRD) patterns were obtained to identify the crystal structures of PtFe_NPs_@PtFe_SAs_‐N‐C, Pt‐N‐C, and Fe‐N‐C. As shown in Figure 1e, five diffraction peaks belonging to the PtFe alloy (JCPDS No. 26–1076) could be found for PtFe_NPs_@PtFe_SAs_‐N‐C. For Pt‐N‐C, a peak at ≈40° was observed, indicating the formation of Pt metal particles (JCPDS No. 04–0802).^[^ ^44^ ^]^For Fe‐N‐C, no Fe crystal information was obtained because of acid treatment. All the samples exhibited a typical peak at ≈26.4°, in line with the (002) facet of graphitic carbon (JCPDS No. 26–1076). Interestingly, in comparison with Pt‐N‐C, the PtFe_NPs_@PtFe_SAs_‐N‐C and Fe‐N‐C samples with Fe species exhibited a substantial enhancement in the sharpness and intensity of the graphitic carbon peak, suggesting that the structural ordering of the carbon matrix was enhanced by Fe species. The degree of graphitization of the samples were characterized using Raman spectroscopy (Figure 1f). At ≈1350 and 1580 cm^−1^, all the samples exhibited D and G bands, corresponding to disordered *sp^3^* carbon and graphitic *sp* ^2^ carbon, respectively. The D‐to‐G band intensity ratios (*I*_D_/*I*_G_) of PtFe_NPs_ @PtFe_SAs_‐N‐C (*I*_D_/*I*_G_ = 0.965) and Fe‐N‐C (*I*_D_/*I*_G_ = 0.908) were lower than that of Pt‐N‐C (*I*_D_/*I*_G_ = 1.028), further confirming that the addition of FeCl_2_ enhanced the graphitization degree of carbon. The decrease in the *I*_D_/*I*_G_ ratio with increasing FeCl_2_ content (Figure S4, Supporting Information) can be attributed to the Fe‐catalyzed graphitization mechanism, which results in ordered graphitized carbon, in line with the XRD results.

The surface chemical states of the PtFe_NPs_@PtFe_SAs_‐N‐C, Fe‐N‐C, and Pt‐N‐C sample were examined using X‐ray photoelectron spectroscopy (XPS). The high‐resolution N 1s spectra in Figure S5 (Supporting Information) show four types of N species in the samples: pyrrolic N and pyridinic N at the edges of the carbon planes, graphitic N in the interior of the graphitic planes, and N‐O bonds. As shown in Figure 1g, the total N contents of PtFe_NPs_@PtFe_SAs_‐N‐C and Fe‐N‐C are 3.40 and 3.95 wt.%, respectively, significantly lower than that of the Pt‐N‐C (6.70 wt.%), indicating that the introduction of Fe species results in a reduction in the N‐doping content. To further investigate this phenomenon, we assessed the graphitic and edge N contents of the different samples and confirmed that all three samples have similar graphitic N contents (1.53–1.58 wt.%), but the edge N contents of PtFe_NPs_@PtFe_SAs_‐N‐C and Fe‐N‐C (1.40 and 2.29 wt.%, respectively) are lower than that of Pt‐N‐C (4.88 wt.%). Evidently, the addition of Fe species led to a decrease in the N‐doping content, which is mainly due to the decrease in edge N incorporation. Thus, the key role of Fe species in decreasing the edge N doping was confirmed (Figure S6, Supporting Information). The edge N content decreased as Fe content increased, whereas the graphitic N content remained relatively stable. This phenomenon can be attributed to the fact that the edge N has a lower bonding strength than the graphitic N in the interior of the graphitic planes, which makes it more susceptible to break off the carbon lattice and volatilize at high temperatures in the presence of Fe species.^[^ ^45^, ^46^ ^]^ The removal of N species partially reconstructs the carbon lattice, further generating more edge defects in the carbon substrate, thereby optimizing the coordination environment of the catalytically active sites.

Based on the aforementioned BET, XRD, Raman, and XPS N 1s analyses, it is reasonable to conclude that the Fe species not only induce more edge defects but also increase the graphitization degree of the carbon support, which can boost the intrinsic catalytic activity and durability of the catalyst.

### 2.2 Electronic and Coordination Structures

**Figure 2:**
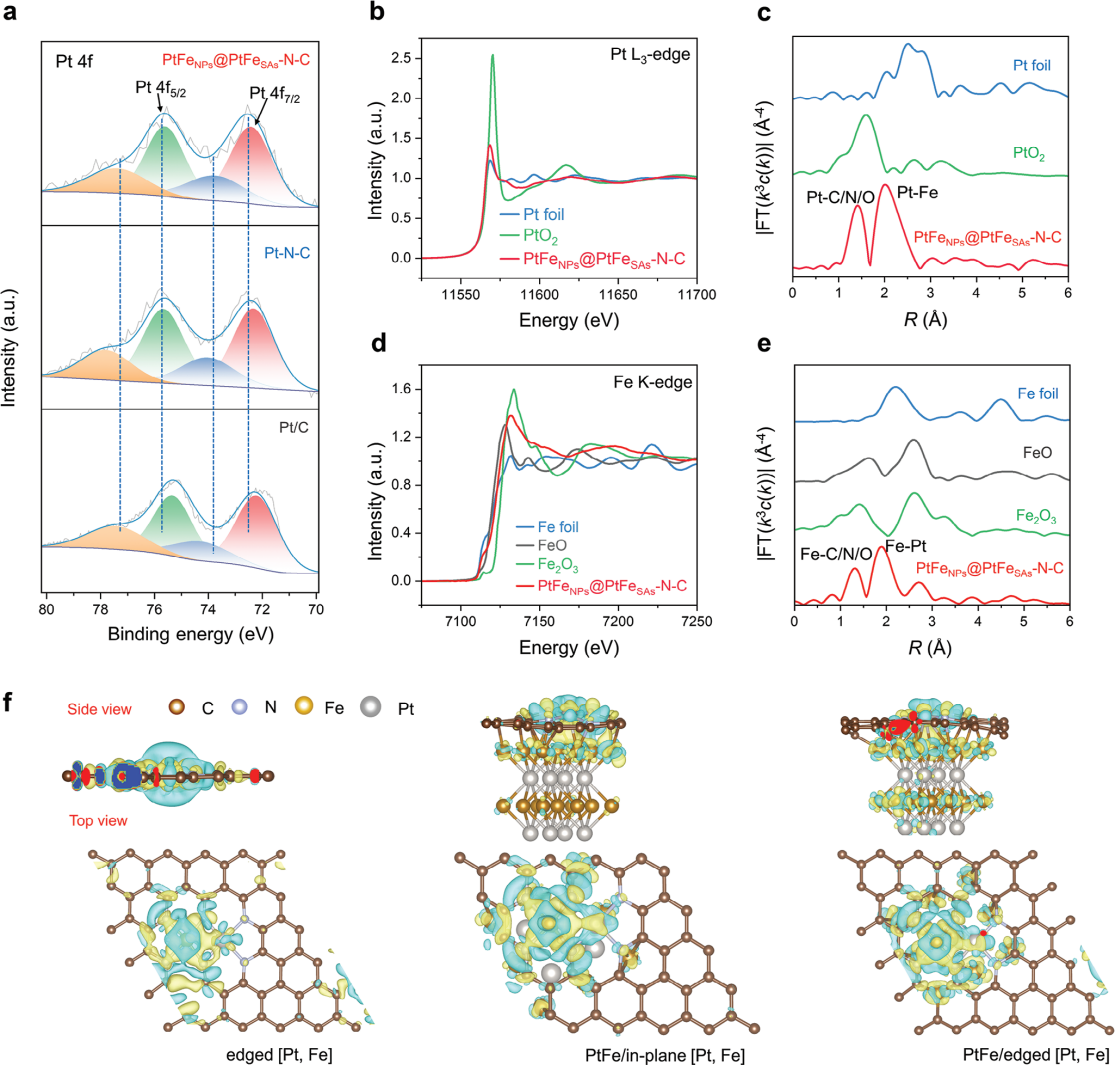
Electronic and atomic structures of PtFe_NPs_@PtFe_SAs_‐N‐C. a) High‐resolution Pt 4f XPS profiles of PtFe_NPs_@PtFe_SAs_‐N‐C, Pt‐N‐C, and commercial Pt/C. b,c) XANES and EXAFS spectra of PtFe_NPs_@PtFe_SAs_‐N‐C at the Pt L_3_‐edge. d,e) XANES and EXAFS spectra of PtFe_NPs_@PtFe_SAs_‐N‐C at the Fe K‐edge. f) Calculated differential charge densities of PtFe/edged [Pt, Fe], PtFe/in‐plane [Pt, Fe], and edged [Pt, Fe]. Cyan and yellow represent electron depletion and accumulation, respectively.

The high‐resolution Pt 4f XPS profile of PtFe_NPs_@PtFe_SAs_‐N‐C exhibits two major peaks at 75.39 and 72.24 eV, corresponding to metallic Pt 4f_5/2_ and Pt 4f_7/2_, representing Pt^0^ in the PtFe nanoparticles (**Figure** **2**a). Additionally, the two peaks located at 74.49 and 77.57 eV indicate the oxidized form of Pt (Pt*^δ^* ^+^), ascribed to single Pt atoms.^[^ ^47^, ^48^ ^]^ Notably, the shift in the Pt^0^ peaks of PtFe_NPs_@PtFe_SAs_‐N‐C to higher binding energies (72.42 and 75.67 eV) relative to those of Pt‐N‐C and commercial Pt/C indicates a strong electronic interaction between single Pt and Fe atoms and the PtFe nanoparticles. This conclusion is further supported by the negative shift (73.78 and 77.42 eV) in the Pt*^δ^* ^+^ peaks of PtFe_NPs_@PtFe_SAs_‐N‐C when compared to the corresponding peaks of Pt‐N‐C and commercial Pt/C.^[^ ^47^, ^48^, ^49^ ^]^ This electronic interaction can modify the charge density of the core metal sites, leading to enhanced catalytic activity.

X‐ray absorption spectroscopy (XAS) was conducted to further probe the coordination environments and chemical states of Pt and Fe, as well as the interaction between the metal sites and carbon substrate in PtFe_NPs_@PtFe_SAs_‐N‐C. The white line intensity of PtFe_NPs_@PtFe_SAs_‐N‐C, as shown in the X‐ray absorption near edge structure spectra (XANES, Figure 2b) of the Pt L_3_‐edge, is stronger than that of the Pt foil, this suggests the transfer of electron from the Pt 5*d* orbitals to the defective N‐doped carbon matrix and Fe 3*d* orbitals, implying that the Pt atoms in PtFe_NPs_@PtFe_SAs_‐N‐C are in the oxidized state. The Fourier transformed (FT) *k^3^*‐weighted extended X‐ray absorption fine structure (EXAFS) spectra reveal a peak of the Pt–Pt bond at 2.00 Å for PtFe_NPs_@PtFe_SAs_‐N‐C, which is shorter than the corresponding peak of the Pt foil (2.51 Å), suggesting the formation of Pt–Fe bonds and electronic interaction between the Pt and Fe atoms and the defective N‐doped carbon matrix in PtFe_NPs_@PtFe_SAs_‐N‐C. Another peak observed at ≈1.41 Å can be ascribed to Pt–N/C/O coordination (Figure 2c). Additionally, the wavelet‐transformed (WT) EXAFS (Figure. S7, Supporting Information) reveals that the corresponding intense peaks of PtFe_NPs_@PtFe_SAs_‐N‐C shifted to a lower **k** value (**k** = 7.5 Å^−1^) compared with those of the Pt foil (**k** = 9.6 Å^−1^), consistent with the existence of light elements (Fe, C, N, and O) around Pt within the robust metal–supporting carbon substrate.^[^ ^23^, ^44^ ^]^

In the Fe K‐edge of PtFe_NPs_@PtFe_SAs_‐N‐C, the near‐edge absorption energy lies between those of Fe and FeO, indicating that the Fe atoms were oxidized (Figure 2d). The strong peak at 1.89 Å in Fourier transform is attributed to the Pt‐Fe alloy scattering; another peak at 1.32 Å is due to the Fe‐C/N/O bonds in the catalyst (Figure 2e). The WT EXAFS shows a strong signal focused at 7.4 Å^−1^, differing from the WT EXAFS of the Fe foil, FeO, and Fe_2_O_3_ (Figure S8, Supporting Information).^[^ ^44^, ^50^ ^]^ The EXAFS structural fitting parameters of Pt and Fe (Figures S9 and S10 and Tables S2 and S3, Supporting Information) confirm that the coordination numbers of both Pt‐C/N/O and Fe‐C/N/O are ≈4, revealing that a single Pt (or Fe) atom was bonded with four light (C/N/O) atoms. Density functional theory (DFT) calculations were performed to investigate the favorable Fe, Pt dual‐atom bonding configurations. Based on the aberration‐corrected HAADF‐STEM and XAS analysis results, we focused on examining four possible coordination environments for Pt and Fe, where they were bonded with C and N atoms at the edges of the defective graphene sheets, including the PtC_3_N_1_‐FeC_3_N_1_, PtC_2_N_2_‐FeC_2_N_2_, PtC_1_N_3_‐FeC_1_N_3_, and PtN_4_‐FeN_4_ models. The edge‐defective carbon mode is represented by the removal of one carbon atom adjacent to the Fe site. The calculated formation energy of PtFeN_4_ is −414.998 eV, lower than those of PtC_3_N_1_‐FeC_3_N_1_, PtC_2_N_2_‐FeC_2_N_2_, and PtC_1_N_3_‐FeC_1_N_3_, suggesting that PtN_4_‐FeN_4_, by bonding of the metal atom to four N atoms in edge‐defective carbon, shows the most stable configuration (Figure S11, Supporting Information).

To gain insights into the interaction mechanism between the PtFe nanoparticles and the edge‐defective carbon embedded with Pt and Fe dual atoms, DFT calculations were performed to analyze the charge density differences for the following three models (Figure S12, Supporting Information): Pt and Fe dual atoms at the edge of the graphene sheet with the PtFe cluster (PtFe/edged [Pt, Fe]), dual‐atom Pt, Fe placed in‐plane on the graphene sheet with the PtFe cluster (PtFe/in‐plane [Pt, Fe]), and dual‐atom Pt, Fe at the edge of the graphene sheet without the PtFe cluster (edged [Pt, Fe]), as shown in Figure 2f. Among these models, PtFe/edged [Pt, Fe] exhibited noticeable and significant electron redistribution. A higher number of electrons originating from the vicinity of the PtFe cluster and central Pt/Fe atoms were observed to migrate toward the edge‐defective graphene region. Consequently, an area with elevated electron density emerged between the edge‐defective graphene sheet and PtFe cluster. This outcome provides compelling evidence for a strong interaction between the PtFe cluster and Pt, Fe dual‐atom‐decorated edge‐defective graphene, consistent with the XPS and XAS results. A comprehensive investigation of the electronic structure and local atomic coordination revealed the coexistence of three types of sites in PtFe_NPs_@PtFe_SAs_‐N‐C, including PtN_4_, FeN_4_, and PtFe nanoparticles, located at the edge of the graphene sheet. Robust electronic interactions between these sites are expected to synergistically enhance the catalytic activity and durability of PtFe_NPs_@PtFe_SAs_‐N‐C.

### 2.3 Electrocatalytic HER Performance

**Figure 3:**
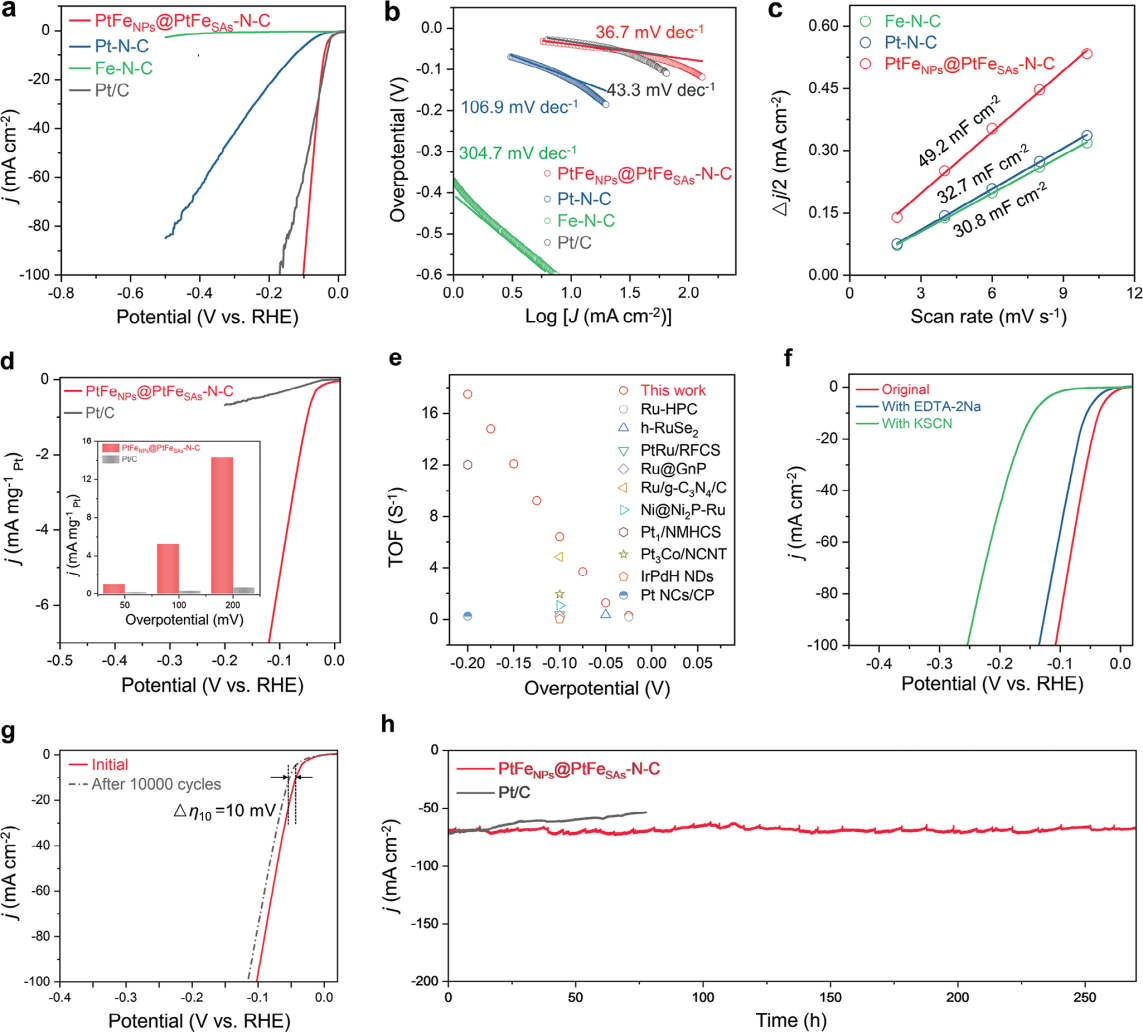
Electrochemical HER performance in 0.5 m H_2_SO_4_. a) LSV curves of PtFe_NPs_@PtFe_SAs_‐N‐C, Pt‐N‐C, Fe‐N‐C, and Pt/C. b) Tafel plots of PtFe_NPs_@PtFe_SAs_‐N‐C, Pt‐N‐C, Fe‐N‐C, and Pt/C. c) Plots of the current density versus scan rate in HER for PtFe_NPs_@PtFe_SAs_‐N‐C, Pt‐N‐C, and Fe‐N‐C. d) Mass activities of PtFe_NPs_@PtFe_SAs_‐N‐C and Pt/C (Inset compares the mass activities at overpotentials of 50, 100, and 200 mV). e) Comparison of the TOF values of PtFe_NPs_@PtFe_SAs_‐N‐C and other recently reported advanced HER catalysts. f) LSV curves of PtFe_NPs_@PtFe_SAs_‐N‐C in 0.5 m H_2_SO_4_ with the addition of KSCN or EDTA‐2Na. g) LSV curves of PtFe_NPs_@PtFe_SAs_‐N‐C before and after 10 000 CV cycles for HER. h) Chronoamperometric curves of PtFe_NPs_@PtFe_SAs_‐N‐C and Pt/C.

The HER activities of the catalysts were first assessed in an N_2_‐saturated 0.5 m H_2_SO_4_ electrolyte using the linear sweep voltammetry (LSV) (**Figure** **3**a). PtFe_NPs_@PtFe_SAs_‐N‐C exhibited a negligible onset potential, close to the thermodynamic potential of HER, and a low overpotential (*η*) of 41 mV at a current density of 10 mA cm^−2^. This overpotential (*η*_10_) value is close to that of Pt/C (20 wt.% Pt, *η*_10_ = 34 mV) and superior to the value of most previously reported Pt‐based HER catalysts (Table S4, Supporting Information). In contrast, the Pt‐N‐C (*η*_10_ = 126 mV) and Fe‐N‐C (*η*_10_ > 500 mV) catalysts exhibited poor activity, this implies that the Pt and Fe species contribute to the HER activity. To gain insights into the HER kinetics, Tafel plots were obtained based on the LSV curves (Figure 3b). PtFe_NPs_@PtFe_SAs_‐N‐C showed the smallest Tafel slope of 36.7 mV dec^−1^ among the different samples, suggesting the most rapid HER kinetics. The Tafel slope suggests that the Volmer–Heyrovsky mechanism occurs in the HER pathway.^[^ ^51^ ^]^ Additionally, the electrochemical impedance spectra (EIS) in Figure S13 (Supporting Information) demonstrated that PtFe_NPs_@PtFe_SAs_‐N‐C had a much smaller charge transfer resistance for HER (R_ct_, ≈18.4 ohm), further confirming the fast HER Kinetic. We further compared the electrochemically active surface areas (ECSAs) of the catalysts, which were assessed from the double‐layer capacitances (*C*_dl_, Figure 3c; Figure S14, Supporting Information). The *C*_dl_ of PtFe_NPs_@PtFe_SAs_‐N‐C (49.2 mF cm^−2^) was significantly larger than those of Pt‐N‐C (30.8 mF cm^−2^) and Fe‐N‐C (32.7 mF cm^−2^), implying that it has a larger ECSA for HER. The large ECSA of PtFe_NPs_@PtFe_SAs_‐N‐C is associated with its defects and porous carbon structure, which exposed abundant active sites for the HER, consistent with the BET results. Given the high cost and scarcity of Pt, the mass activity was normalized to the loaded Pt mass (Figure 3d). PtFe_NPs_@PtFe_SAs_‐N‐C delivered mass activities of 1.01, 5.21, and 14.30 mA mg^−1^_Pt_ at overpotentials of 50, 100, and 200 mV, respectively. These activities are ≈8, 15, and 20 times greater than those of Pt/C, respectively, demonstrating that PtFe_NPs_@PtFe_SAs_‐N‐C can be significantly economical. To investigate the intrinsic electrocatalytic activity, the turnover frequency (TOF) of PtFe_NPs_@PtFe_SAs_‐N‐C was calculated according to metal loading (Figure 3e). Notably, the TOF of PtFe_NPs_@PtFe_SAs_‐N‐C was higher than that of most reported noble‐based HER electrocatalysts.^[^ ^19^, ^52^, ^53^ ^]^ The effect of the amount of Fe salt on the HER activity was also investigated. Fe‐100‐Pt‐N‐C and Fe‐500‐Pt‐N‐C exhibited higher overpotentials at 10 mA cm^−2^, which can be ascribed to the variation in the edge‐rich defects and graphitization degree of the carbon (Figure S15, Supporting Information).

The strong catalytic synergy between the PtFe nanoparticles and edge‐hosted Pt and Fe atoms in PtFe_NPs_@PtFe_SAs_‐N‐C was confirmed through poisoning experiments using ethylenediaminetetraacetic acid disodium (EDTA‐2Na) and potassium thiocyanide (KSCN). Previous studies have shown that EDTA‐2Na selectively coordinates with single metal atoms, whereas KSCN inhibits the activity of both metal nanoparticles and single metal atoms.^[^ ^49^ ^]^ PtFe_NPs_@PtFe_SAs_‐N‐C with EDTA‐2Na showed a significant decrease in the HER activity, and it was deactivated when added with KSCN (Figure 3f), indicating the necessity of both PtFe nanoparticles and edge‐defective carbon embedded with dual Pt and Fe atoms for high catalytic activity.

To evaluate the stability of PtFe_NPs_@PtFe_SAs_‐N‐C in the HER, polarization curves were obtained, which revealed a slight negative shift in the potential of 10 mV at 10 mA cm^−2^ after 10000 cyclic voltammetry (CV) sweeps (Figure 3g). Moreover, the current density (70 mA cm^−2^) at the constant overpotential showed negligible decrease over 270 h of catalytic HER (Figure 3h). In contrast, Pt/C showed an evident decrease in the activity throughout the 77 h test owing to weak interactions between the carbon substrate and Pt particles, resulting in the detachment of the Pt particles. The robustness of the PtFe_NPs_@PtFe_SAs_‐N‐C electrocatalyst was confirmed through SEM and TEM characterization after the durability test. As shown in Figure S16 (Supporting Information), the porous structure of the PtFe_NPs_@PtFe_SAs_‐N‐C catalyst was well preserved, with no significant particle aggregation observed after long‐term electrolysis. Additionally, XPS analysis revealed that the primary elements retained their initial chemical states, highlighting the catalyst's durability and stability under operational conditions (Figure S17, Supporting Information). The remarkable stability of PtFe_NPs_@PtFe_SAs_‐N‐C in the HER can be attributed to the robust EMSI between the PtFe nanoparticles and edge‐defect‐hosted single Pt and Fe atoms, which may lead to a reduction in metal site dissolution and/or agglomeration. In addition, the high graphitization degree of the carbon support in PtFe_NPs_@PtFe_SAs_‐N‐C significantly enhanced the corrosion resistance and electronic conductivity of the catalyst.^[^ ^54^ ^]^

### 2.4 Performance of an FSAAH with PtFe_NPs_@PtFe_SAs_‐N‐C Cathode

**Figure 4:**
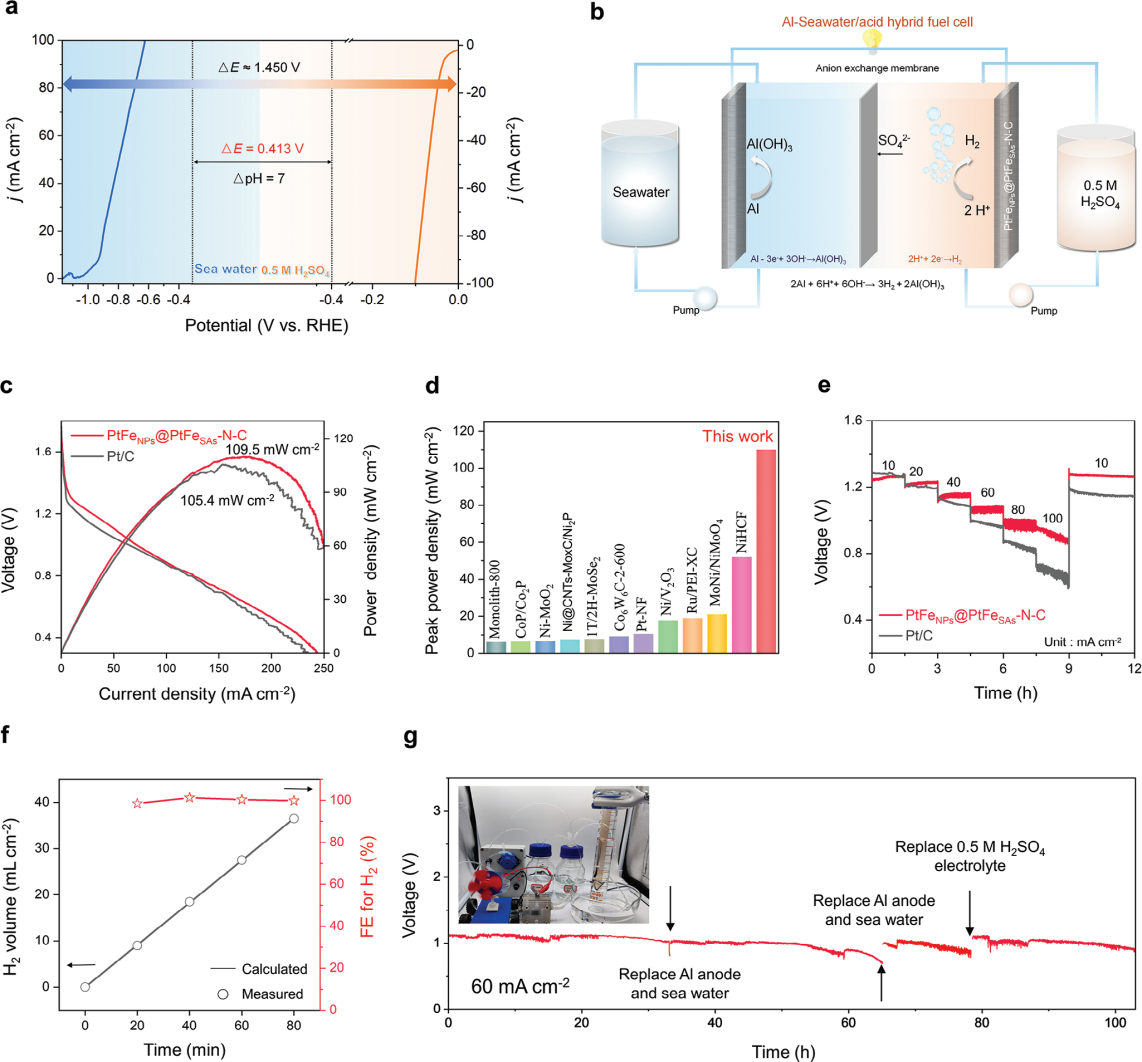
Performance of a flow seawater‐Al/acid hybrid fuel cell (FSAAH). a) Al oxidation polarization curve in seawater and HER polarization curve of PtFe_NPs_@PtFe_SAs_‐N‐C in 0.5 m H_2_SO_4_, and the voltage generated from the pH difference between the seawater and 0.5 m H_2_SO_4_. b) Schematic illustration of the PtFe_NPs_@PtFe_SAs_‐N‐C catalyst‐based FSAAH. c) Discharge polarization curves and power density curves of PtFe_NPs_@PtFe_SAs_‐N‐C FSAAH and Pt/C FSAAH. d) Comparison of recently reported advanced catalyst‐based Al/Mg seawater batteries. e) Chronopotentiometric response curves of the PtFe_NPs_@PtFe_SAs_‐N‐C FSAAH at different current densities. f) Measured and calculated H_2_ production and H_2_ Faradaic efficiency of the PtFe_NPs_@PtFe_SAs_‐N‐C FSAAH at a current density of 60 mA cm^−2^. g) Chronopotentiometric response curve of the PtFe_NPs_@PtFe_SAs_‐N‐C FSAAH device at a current density of 60 mA cm^−2^ (inset shows a digital photograph of a PtFe_NPs_@PtFe_SAs_‐N‐C FSAAH powering a windmill, accompanied by hydrogen gas generation).

Hydrogen production via direct seawater electrolysis without further treatment is gaining increasing interest because of the abundance and availability of seawater resources on Earth. However, the slow kinetics of the OER and HER in low‐conductivity neutral seawater, along with the poisoning of the catalyst by impurities present in seawater, often hinder seawater electrolysis from satisfying the hydrogen production rate and operational lifetime requirements for industrial‐scale applications.^[^ ^55^, ^56^, ^57^ ^]^ Inspired by the outstanding HER performance of PtFe_NPs_@PtFe_SAs_‐N‐C, we developed a novel FSAAH device. The pH difference between seawater and the acid electrolyte enhanced its performance, resulting in a high theoretical voltage and output energy density compared with those of conventional metal–seawater batteries (**Figure** **4**a). In this setup, an Al plate served as the anode for Al oxidation in a flowing simulated seawater electrolyte, PtFe_NPs_@PtFe_SAs_‐N‐C was employed as the cathode catalyst for the HER in a flowing acid electrolyte (0.5 m H_2_SO_4_), and between the anode and cathode compartments, an anion exchange membrane separated them (Figure 4b). PtFe_NPs_@PtFe_SAs_‐N‐C FSAAH achieved a high open‐circuit voltage (*V*_oc_) of 1.50 V and output a maximum power density of 109.5 mW cm^−2^ at the current density of 179 mA cm^−2^ (Figure 4c; Figure S18, Supporting Information). This peak power density surpasses those of the Pt/C FSAAH (105.4 mW cm^−2^) and most of the conventional Al or Mg/seawater batteries (Figure 4d; Table S5, Supporting Information). Moreover, the galvanostatic discharge curve of the FSAAH with PtFe_NPs_@PtFe_SAs_‐N‐C (Figure 4e) indicated excellent rate capability and reversible performance. The FSAAH exhibited discharge voltages of ∼1.25, 1.21, 1.15, 1.10, 1.05, and ≈0.99 V at discharge current densities of 10, 20, 40, 60, 80, and 100 mA cm^−2^, respectively. Importantly, the discharge voltage could be completely restored when the current density was returned to 10 mA cm^−2^.

Moreover, the H_2_ product was collected in situ using the drainage method during discharge (Figure S19 and Video S1, Supporting Information), at the current density of 60 mA cm^−2^, achieving a remarkable H_2_ production rate of 271.6 L m^−2^ h^−1^ and a high Faradaic efficiency of ≈99.0% (Figure 4f). Furthermore, at a high discharge current density of 60 mA cm^−2^, the PtFe_NPs_@PtFe_SAs_‐N‐C FSAAH device exhibited no notable decrease in voltage upon continuous operation for over 100 h (Figure 4g), as long as the Al anode and electrolyte supply were maintained, implying the robust durability of this FSAAH. To validate its practical applicability, a miniature windmill was successfully powered with a single PtFe_NPs_@PtFe_SAs_‐N‐C FSAAH, and H_2_ bubbles were visibly observed at the cathode side and collected using the drainage method (Video S1, Supporting Information). These results strongly confirm the feasibility of developing an FSAAH with a high H_2_ production efficiency, high output power density, and robust durability, demonstrating its great potential in many fields, such as the realization of efficient energy‐conversion devices for the utilization of the infinite seawater resource.

### 2.5 ORR and ZAB Performance

**Figure 5:**
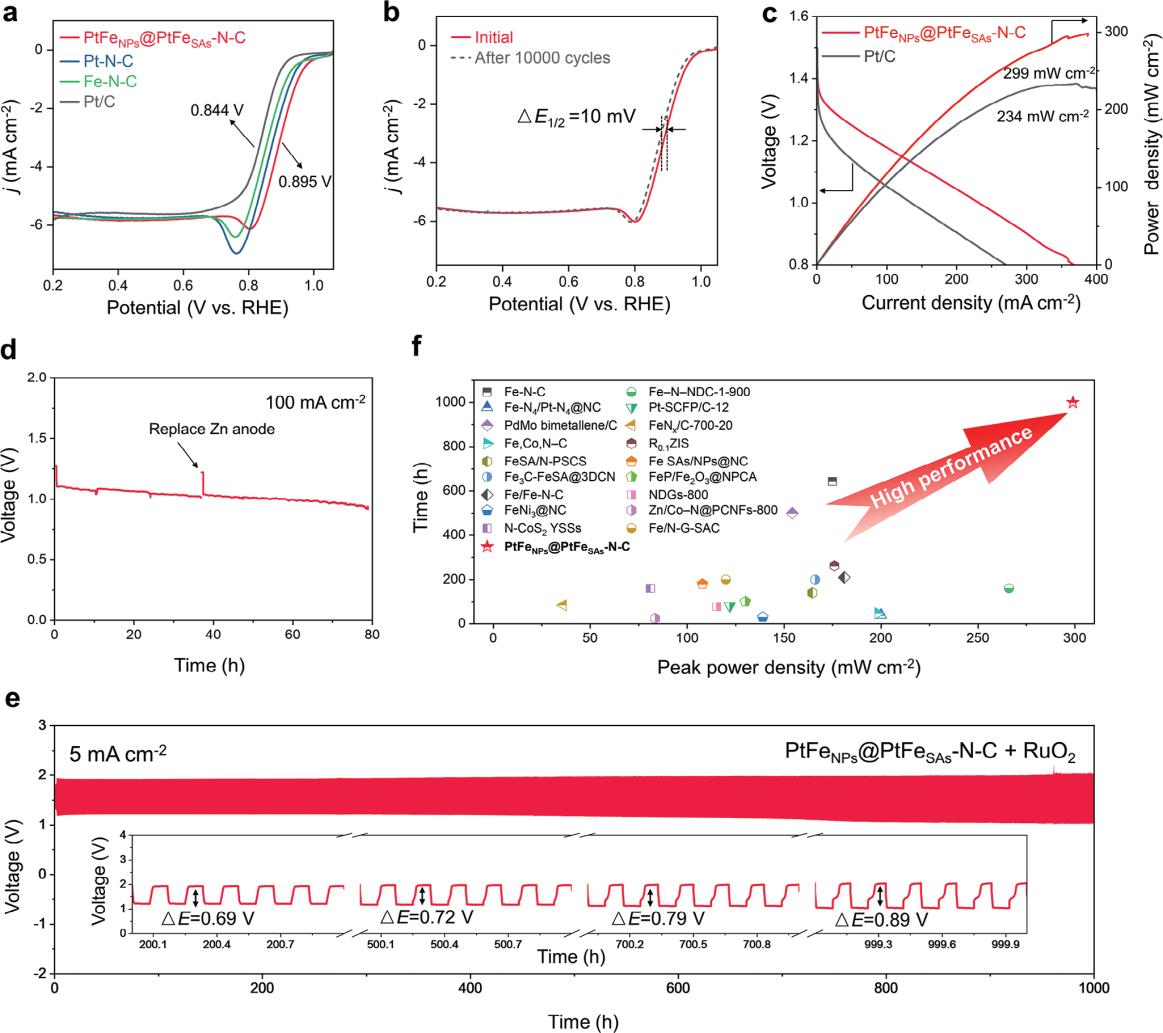
ORR and flow Zn–air battery (ZAB) performance. a) RDE polarization curves of PtFe_NPs_@PtFe_SAs_‐N‐C, Pt‐N‐C, Fe‐N‐C, and Pt/C. b) ORR polarization curves of PtFe_NPs_@PtFe_SAs_‐N‐C before and after 10 000 cycles. c) Discharge polarization curves and power density curves of PtFe_NPs_@PtFe_SAs_‐N‐C ZAB and Pt/C ZAB. d) Chronopotentiometric response curve of PtFe_NPs_@PtFe_SAs_‐N‐C ZAB at 100 mA cm^−2^. e) Comparison of the peak power density and discharge–charge cycling time at 5 or 10 mA cm^−2^ for ORR catalyst‐based Zn–air batteries. f) Galvanostatic discharge–charge cycling curve of PtFe_NPs_@PtFe_SAs_‐N‐C + RuO_2_ at 5 mA cm^−2^ (inset shows the enlarged discharge–charge curve).

The ORR performance of the catalysts was evaluated using the rotating disk electrode (RDE) technique in a 0.1 m KOH electrolyte (Figure S20, Supporting Information). The slight peaks observed in the polarization curves of PtFe_NPs_@PtFe_SAs_‐N‐C, Pt‐N‐C, and Fe‐N‐C before reaching the limited current density may be related to the removal of adsorbed oxygen molecules from the surface during the reaction process.^[^ ^58^, ^59^, ^60^ ^]^ Notably, PtFe_NPs_@PtFe_SAs_‐N‐C exhibited the highest half‐wave potential (*E*_1/2_) of 0.895 V (**Figure** **5**a; Table S6, Supporting Information); the *E*_1/2_ values of Fe‐N‐C, Pt‐N‐C, and Pt/C were 0.864, 0.881, and 0.844 V, respectively. This result indicates that the presence of both Pt and Fe species significantly improves the ORR activity, as further demonstrated by poisoning experiments using EDTA‐2Na and KSCN (Figure S21a, Supporting Information). The kinetic current density (*J*_k_, Figure S21b, Supporting Information) of PtFe_NPs_@PtFe_SAs_‐N‐C (41.1 mA cm^−2^) was higher than those of Pt‐N‐C (15.4 mA cm^−2^), Fe‐N‐C (8.48 mA cm^−2^), and Pt/C (4.52 mA cm^−2^), revealing its more favorable kinetics. The fast ORR kinetics were also highlighted through the Tafel slope analysis (Figure S21c, Supporting Information). A lower Tafel slope of 86.4 mV dec^−1^ was obtained for PtFe_NPs_@PtFe_SAs_‐N‐C compared with those of Fe‐N‐C (96.4 mV dec^−1^) and Pt‐N‐C (89.7 mV dec^−1^). Additionally, the EIS analysis further confirmed the rapid ORR kinetics for PtFe_NPs_@PtFe_SAs_‐N‐C, as evidenced by its minimum charge transfer resistance (Rct, ≈135 ohm) compared to the other counterparts (Figure S22, Supporting Information). Moreover, PtFe_NPs_@PtFe_SAs_‐N‐C exhibited the largest *C*_dl_ of 28.6 mF cm^−2^ (Figure S23, Supporting Information). Control experiments for the catalysts with various amounts of Fe demonstrated that Fe‐300‐Pt‐N‐C exhibits the best ORR catalytic activity (Figure S24, Supporting Information).

We investigated the ORR pathways and H_2_O_2_ yields of PtFe_NPs_@PtFe_SAs_‐N‐C. The number of electrons transferred (*n*) for PtFe_NPs_@PtFe_SAs_‐N‐C was determined to be close to four from the Koutecky–Levich (K–L) plot (Figure S25a,b, Supporting Information), indicating that the ORR follows a four‐electron reaction process in the reduction of O_2_ to H_2_O. Further, the H_2_O_2_ yield was monitored using the rotating ring‐disk electrode (RRDE) method. As shown in Figure S25c (Supporting Information), the H_2_O_2_ yield is <3% and the *n* value is 3.94–3.98 at 0.2–0.8 V versus the reversible hydrogen electrode (RHE). In contrast, Pt‐N‐C, Fe‐N‐C, and Pt/C exhibit higher H_2_O_2_ yields of up to 9% and relatively lower n values of 3.75–3.97 at 0.2–0.8 V versus RHE (Figure S26, Supporting Information). These results demonstrate that the ORR on PtFe_NPs_@PtFe_SAs_‐N‐C follows a four‐electron transfer pathway with a high selectivity for ORR. Furthermore, PtFe_NPs_@PtFe_SAs_‐N‐C has excellent methanol tolerance (Figure S27a, Supporting Information), as evidenced by the negligible change in current following the introduction of 1.0 m methanol. In contrast, Pt/C exhibited a significant decline in current owing to the competing methanol oxidation reaction. In addition, after 10 000 CV cycles, the ORR polarization curves (Figure 5b) of PtFe_NPs_@PtFe_SAs_‐N‐C display only a minor negative shift (10 mV) in the *E*_1/2_, implying that PtFe_NPs_@PtFe_SAs_‐N‐C has good long‐term durability. Importantly, PtFe_NPs_@PtFe_SAs_‐N‐C could operate continuously for 220 h and maintain 97.6% current, indicating its high stability (Figure S27b, Supporting Information). Post‐characterizations (XPS, SEM, TEM) of the PtFe_NPs_@PtFe_SAs_‐N‐C catalyst after the durability test show that the catalyst retained its initial state, further confirming its stability (Figures S28 and S29, Supporting Information).

The excellent ORR activity of PtFe_NPs_@PtFe_SAs_‐N‐C renders it a suitable air cathode catalyst in a ZAB, as shown in Figure S30 (Supporting Information). In addition to exhibiting a high open‐circuit voltage of 1.465 V (Figure S31, Supporting Information), the PtFe_NPs_@PtFe_SAs_‐N‐C ZAB presented excellent discharge behavior, with a peak power density of 298.0 mW cm^−2^ at a current density of 388.1 mA cm^−2^ (Figure 5c), surpassing that of the Pt/C‐based ZAB (233.7 mW cm^−2^). We also examined the rate capability of the PtFe_NPs_@PtFe_SAs_‐N‐C ZAB at different current densities. Figure S32 (Supporting Information) shows that the PtFe_NPs_@PtFe_SAs_‐N‐C ZAB exhibits a small voltage drop from 1.31 V at 10 mA cm^−2^ to 1.03 V at 200 mA cm^−2^. The discharge voltage resumes reversibly once the current density is decreased to 10 mA cm^−2^, which suggests its excellent discharge rate performance. At a high current density of 100 mA cm^−2^, the PtFe_NPs_@PtFe_SAs_‐N‐C ZAB could discharge a voltage of up to 1 V for 40 h until the Zn anode was almost completely consumed (Figure 5d). When the Zn anode was replaced, the battery could operate at the same performance level (the discharge voltage returned to 1 V) for 40 h, indicating the durability of PtFe_NPs_@PtFe_SAs_‐N‐C. We further used a combination of PtFe_NPs_@PtFe_SAs_‐N‐C and RuO_2_ as cathode catalysts for galvanostatic discharge–charge cycling tests (Figure 5f). Notably, the battery could be stably cycled for 1000 h at 5 mA cm^−2^; the initial charge–discharge voltage gap (▵*E*) was only 0.69 V, and the energy efficiency was as high as 63.9% (the inset of Figure 5f). With continuous flowing and cycling for 1000 h, the ▵*E* increased to only 0.89 V with a high energy efficiency of 54.6%, demonstrating superior cycling stability. The high output power density and long‐life performance of the PtFe_NPs_@PtFe_SAs_‐N‐C ZAB evidently indicate that it outperforms most of the recently reported electrocatalyst‐based Zn–air batteries (Figure 5e; Table S7, Supporting Information).

### 2.6 Density Functional Theory Calculations

**Figure 6:**
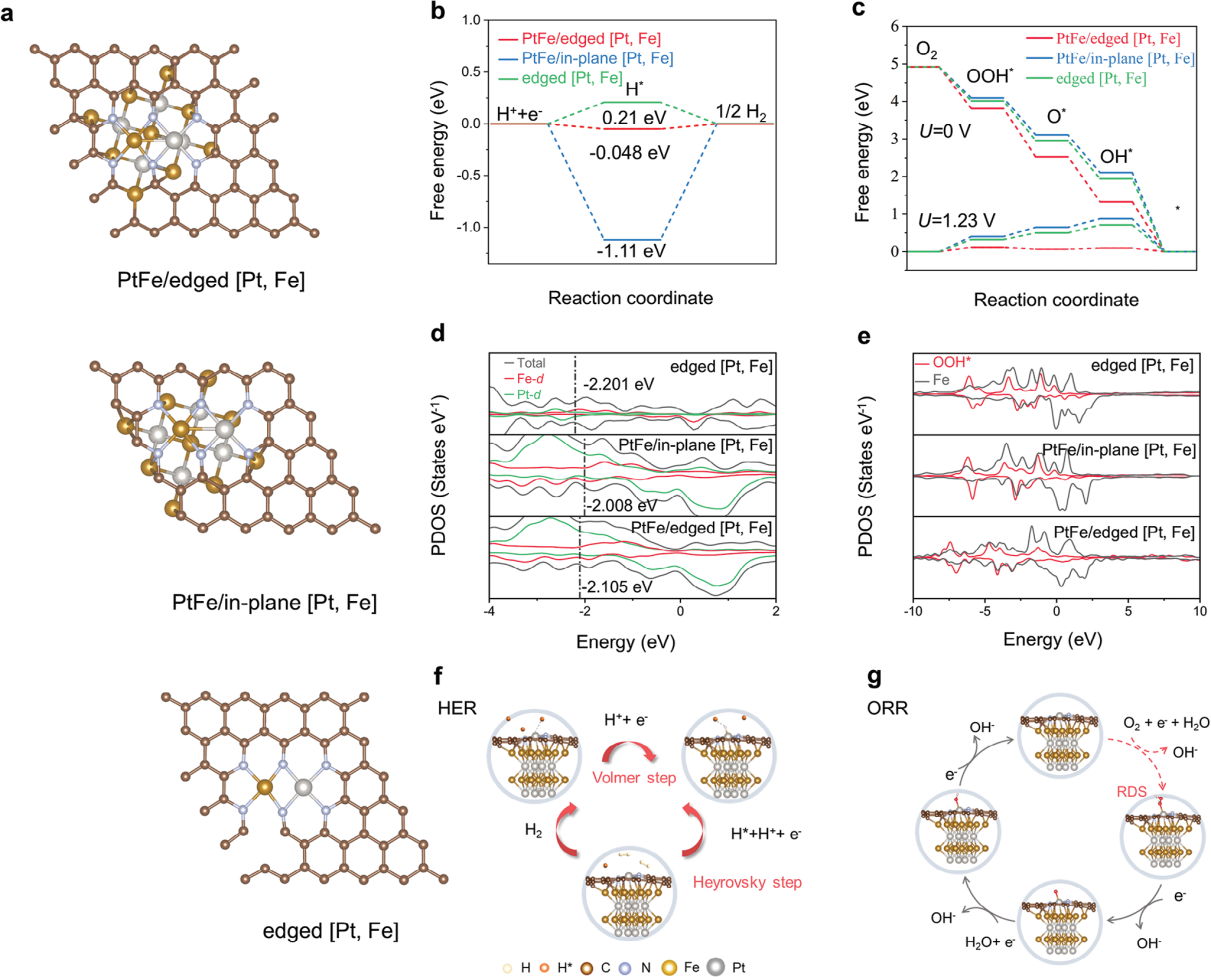
DFT calculations. a) Surface structures of PtFe/edged [Pt, Fe], PtFe/in‐plane [Pt, Fe], and edged [Pt, Fe]. b) Gibbs free energy diagrams of the HER. c) Gibbs free energy diagrams of the ORR. d) Projected density of states of total, Pt‐*d*, Fe‐*d* in PtFe/edged [Pt, Fe], PtFe/in‐plane [Pt, Fe], and edged [Pt, Fe]. e) Projected density of states of OOH*, Fe‐*d* in PtFe/edged [Pt, Fe], PtFe/in‐plane [Pt, Fe], and edged [Pt, Fe]. f,g) Proposed HER and ORR pathways, respectively, on PtFe_NPs_@PtFe_SAs_‐N‐C.

To further clarify the influence of the PtFe nanoparticles and edge‐defective carbon‐embedded with Pt and Fe dual atoms on the HER and ORR activities of PtFe_NPs_@PtFe_SAs_‐N‐C, DFT calculations were performed on the PtFe/edged [Pt, Fe], PtFe/in‐plane [Pt, Fe], and edged [Pt, Fe] models (**Figure** **6**a). Gibbs free energy of H* (∆*G*_H*_) is regarded as a vital criterion to assess the HER activity of electrocatalysts (Figure 6b). The ∆*G*_H_* value was determined to be greater than zero (positive) for the edged [Pt, Fe], implying that the edged [Pt, Fe] has weak binding ability to H*. Consequently, a higher energy input is necessary to activate the Volmer step, which reduces the overall catalytic efficiency of the catalyst. For the PtFe/edged [Pt, Fe] and PtFe/in‐plane [Pt, Fe] models, the ∆*G*_H*_ values fall below zero, indicating that the Volmer step is favored. However, the strong adsorption of H* species onto PtFe/in‐plane [Pt, Fe] may hinder H* desorption. Among these models, PtFe/edged [Pt, Fe] demonstrated the most favorable desorption–adsorption strength toward H* species, as indicated by the lowest ∆*G*_H*_ value of −0.05 eV. This favorable desorption–adsorption of H* ensures a dynamic balance between proton capture and hydrogen release, thereby accelerating the HER, which was also confirmed by the optimized Fe‐H bond length of PtFe/edged [Pt, Fe] (Figure S33, Supporting Information). The difference in the *d*‐band center can further explain the difference in the H* adsorption strength. The partial density of states (PDOS) is shown in Figure 6d. The energies of the *d*‐band centers of PtFe/edged [Pt, Fe], PtFe/in‐plane [Pt, Fe], and edged [Pt, Fe] were −2.105, −2.008, and −2.201 eV, respectively, revealing a neither too strong nor too weak binding strength of the adsorbed H* species on PtFe/edged [Pt, Fe]. Charge density difference analyses confirmed a moderate interaction between the PtFe/edged [Pt, Fe] and adsorbed H* (Figure S34, Supporting Information). Therefore, the interaction of PtFe nanoparticles with edge‐defect carbon embedded with Pt and Fe dual atoms can regulate the *d*‐band center, thereby yielding optimal H* adsorption–desorption and enhanced HER activity. Based on the Tafel and DFT calculation results, the HER on PtFe/edged [Pt, Fe] tended to follow the Volmer–Heyrovsky mechanism, as shown in Figure 6f.

For the ORR, based on the RDE and RRDE results, DFT calculations were conducted based on the associative mechanism with a four‐electron pathway (Figure S35, Supporting Information). The free‐energy diagrams of the ORR for the three models are shown in Figure 6c, which exhibit a similar downhill energy pathway at *U* = 0 V, indicating a spontaneous exothermic process. At *U* = 1.23 V, the energy of the step involving O* → OOH* is notably higher than those of the other steps across the three models, suggesting that the hydrogenation of O* to form OOH* is the rate‐determining step (RDS). For the PtFe/edged [Pt, Fe], the energy barrier for RDS has the lowest thermodynamic overpotential (*η* = 0.12 V) (*η* = 0.40 V for PtFe/in‐plane [Pt, Fe] and *η* = 0.32 V for edged [Pt, Fe]), highlighting the critical role of PtFe nanoparticles and edge defect carbon‐embedded with Pt and Fe atoms in facilitating the ORR. In addition, the shorter Fe‐O bond of the OOH* adsorbed on the PtFe/edged [Pt, Fe] site (1.813 Å) than in the cases of the PtFe/in‐plane [Pt, Fe] site (1.855 Å) and edged [Pt, Fe] (1.851 Å) suggests the stronger adsorption of OOH* to the former (Figure S36, Supporting Information). Furthermore, PDOS calculations (Figure 6e) revealed that more hybridization occurred between the Fe sites and OOH* intermediates near the Fermi level on the PtFe/edged [Pt, Fe], resulting in stronger adsorption of OOH* intermediates, thus favoring the ORR performance. Meanwhile, charge density differences and Bader charge analyses (Figure S37, Supporting Information) revealed that greater charge transfer occurred between the PtFe/edged [Pt, Fe] site and OOH* intermediates, which strengthened the interaction between the two. The strong interaction between the PtFe nanoparticles and edge‐defective carbon embedded with dual Pt and Fe atoms leads to more potent adsorption of OOH* intermediates, thereby contributing to enhanced ORR performance. Based on these results, the ORR pathway of PtFe_NPs_@PtFe_SAs_‐N‐C is revealed, as shown in Figure 6g.

## 3 Conclusion

We prepared a PtFe_NPs_@PtFe_SAs_‐N‐C hybrid material consisting of dual Pt and Fe atoms and PtFe nanoparticles on a modified N‐doped carbon support. Comprehensive structural characterization confirmed that well‐balanced edge defects and graphitized carbon were achieved in PtFe_NPs_@PtFe_SAs_‐N‐C. The abundant edges and porosity of carbon were induced by Fe etching, and the high graphitization degree of carbon was derived from the Fe‐catalyzed mechanism. XPS and XAS analyses revealed that electron transfer occurred between the PtFe nanoparticles and the edge‐defective carbon embedded with Pt and Fe dual atoms, resulting in strong metal–support interactions. Consequently, PtFe_NPs_@PtFe_SAs_‐N‐C exhibited excellent activity and long durability in HER and ORR. DFT calculations revealed that the strong EMSI optimized the adsorption–desorption of hydrogen/oxygen intermediates in HER/ORR and reduced the reaction energy barriers, resulting in enhanced HER and ORR activities. The PtFe_NPs_@PtFe_SAs_‐N‐C cathode catalyst delivered a high‐power density and exhibited long‐term durability in novel flow seawater‐Al/acid hybrid fuel cells and flow Zn–air batteries. More interestingly, the flow seawater‐Al/acid hybrid fuel cell utilizes abundant seawater as the anode electrolyte without further power grids, enabling on‐site, self‐powered hydrogen production, indicating its potential for efficient energy conversion and the utilization of infinite seawater resources. Therefore, this study provides key insights for designing carbon‐supported Pt‐based materials with balanced defective graphitization and strong EMSI for various catalytic and advanced energy applications.

## Conflict of Interest

The authors declare no conflict of interest.

## Supporting information

Supporting Information

Supplemental Video 1

## Data Availability

The data that support the findings of this study are available from the corresponding author upon reasonable request.
