# Supplementary material for: Balancing Edge Defects and Graphitization in a Pt–Fe/Carbon Electrocatalyst for High‐Power‐Density and Durable Flow Seawater‐Al/Acid Hybrid Fuel Cells and Zn–Air Batteries
Source: Adv Sci (Weinh). 2024 Sep 5;11(41):2308923. doi: 10.1002/advs.202308923 (PMC11538727; doi:10.1002/advs.202308923)
Supplement: Supplementary file 1 — Supporting Information [file ADVS-11-2308923-s002.docx]

Balancing Edge Defects and Graphitization in a Pt-Fe/Carbon Electrocatalyst for High-power-Density and Durable Flow Seawater-Al/Acid Hybrid Fuel Cells and Zn-air Batteries

Hao Li^a, b, #^, Mengtian Zhang^a#^, Mi Wang^a, b,*^, Minghao Du^a, b^, Zijian Wang ^a, b^, Yongxing Zou^a, b^, Guangxing Pan ^a, b^, Jiaheng Zhang^a, b,*^

^a^ Sauvage Laboratory for Smart Materials, Harbin Institute of Technology (Shenzhen), Shenzhen, 518055, China

^b^ Research Centre of Printed Flexible Electronics, School of Materials Science and Engineering, Harbin Institute of Technology, Shenzhen 518055, China

^*^ Corresponding authors: [wangmi@hit.edu.cn](mailto:wangmi@hit.edu.cn) (M. Wang); zhangjiaheng@hit.edu.cn (J.H. Zhang)

^#^ These authors contributed equally to this work.

1. **Experimental Section**

Material preparation

Synthesis of PtFe_NPs_@PtFe_SAs_-N-C: First, tannic acid (1.5 g), ZnCl_2_ (0.8 g), urea (2.4 g), NaCl (80 g), FeCl_2_·4H_2_O (300 mg), and K_2_PtCl_4_ (30 mg) were dispersed in an aqueous solution (300 mL) containing Tris (1.1 g) and stirred. Next, the mixed solution was spray-dried using N_2_ as the carrier gas. Then, the resultant powder was subjected to a 5-hour heat treatment at 300 °C in the air. Then, under Ar atmosphere, the powder was annealed at 600 °C for 2 h and then at 900 °C for 2 h. Subsequently, the obtained product was etched with 1 M HCl solution to remove unstable metal species, followed by a thorough washing with deionized water and finally vacuum drying overnight at 60℃. The resulting product was named as PtFe_NPs_@PtFe_SAs_-N-C or Fe-300-Pt-N-C.

Synthesis of Fe-x-Pt-N-C and Fe-N-C: The method for synthesizing Fe-x-Pt-N-C (where x represents the amount of FeCl_2_·4H_2_O) is analogous to that of Fe-300-Pt-N-C except that the amount of FeCl_2_·4H_2_O was varied as 0, 100, and 500 mg. The resulting powders were named as Pt-N-C, Fe-100-Pt-N-C, and Fe-500-Pt-N-C. The procedure for synthesizing Fe-N-C is analogous to that of Fe-300-Pt-N-C, with the exception of not adding K_2_PtCl_4_.

Physical characterization: The scanning electron microscope (SEM, ZEISS Sigma 300), transmission electron microscope (TEM, Tecnai F20, 200 k) and aberration-corrected high-angle annular dark-field scanning transmission electron microscope (HAADF-STEM, FEI Theims Z) were used to analyze microstructural morphology of the samples. The PANalytical/Aeris X-ray diffractometer was used to analyze the crystalline structure of the samples. Raman spectra were collected using a 532 nm excitation laser from a Renishaw/inVia Qontor. The Micromeritics ASAP 2460 BET surface area analyzer was used to recorded Nitrogen adsorption–desorption isotherms. The ESCALAB 250Xi XPS system was used to collect X-ray photoelectron spectra (XPS). The extended X-ray absorption fine structure (EXAFS) data for Fe K-edge and Pt L_3_-edge were obtained at the Shanghai Synchrotron Radiation Facility.

Electrochemical tests and measurements: The CHI 760E electrochemical workstation was used to evaluate the HER and ORR activities in a three-electrode cell setup. This setup includes the catalyst-modified working electrode, a graphite rod counter electrode, and a reference electrode. In case of 0.5 M H_2_SO_4_ electrolyte, a saturated Ag/AgCl (sat. KCl) reference electrode, while the Hg/HgO reference electrode was used in 0.1 M KOH. The working electrode was prepared by dispersing the catalyst in a mixture of 5 wt% Nafion solution (50 μL), ethanol (30 μL), and water (420 μL) through 30 minutes of sonication. Subsequently, the catalyst link was deposited onto a glassy carbon electrode to achieve a mass loading of 0.85 mg cm^−2^.

For evaluating the HER and ORR activities, linear sweep voltammetry (LSV) was performed at 5 mV s^−1^ in a N_2_-saturated 0.5 M H_2_SO_4_ solution and O_2_-saturated 0.1 M KOH solution, respectively. RRDE measurements were performed at rotating speeds ranging from 625 to 2500 rpm at 5 mV s^−1^. EIS measurement involved sweeping the frequency from 100 kHz to 0.1 Hz. And the ECSA values of the samples were determined by analyzing the slope of the charging current versus scan rate, which corresponds to the electrochemical double-layer capacitance (C_dl_). All potentials were normalized to the RHE potentials using the following equation:

$\text{E}_{RHE}\text{= 0.197 +}\text{ }\text{0.059 × pH}\text{ }\text{+ }\text{E}_{Ag/AgCl}$ (1)

$\text{E}_{RHE}\text{= 0.098 +0.059 × pH}\text{ }\text{+}\text{ }\text{E}_{Hg/HgO}$ (2)

The K–L analysis was conducted utilizing the RDE measurement, and the electron transfer number (n) and percentage of H_2_O_2_ were acquired through the RRDE test.

Assembly and electrochemical test of FSAAH: The measurements on FSAAH were conducted with saturated Ar atmosphere in both electrolyte chambers at room temperature. Titanium mesh (1 mg cm^−2^) modified with PtFe_NPs_@PtFe_SAs_-N-C or Pt/C-was served as the cathode to generate hydrogen in a acid electrolyte (0.5 M H_2_SO_4_), and a Al plate was used as the anode to realize Al oxidation in simulated seawater (0.5 M NaCl). The cathodic and anodic chambers were separated with an anion-exchange membrane. The flow electrolytes at the rate of approximately 15 mL min^−1^ were filled with the chambers (the electrolyte was supplied from two sealed containers). Before operating the FSAAH, nitrogen gas was introduced into the electrolyte to remove any remaining oxygen. The CHI 760E electrochemical workstation was used to record the LSV curves from 1.75 to 0.4 V at 5 mV s^−1^. Rate discharge curves and long-term durability tests were conducted on a Land CT3001A multichannel battery testing system.

Assembly and electrochemical test of the ZAB: The ZABs were custom-built using a Zn plate as the anode and carbon cloth (1 cm^−2^) coated with the catalyst as the air cathode. The electrolyte was a solution of 0.2 M zinc acetate and 6.0 M KOH. The flow electrolyte at the rate of approximately 15 mL min^−1^ was filled with the chamber. The CHI 760E electrochemical workstation was used to record the LSV curves from 1.5 to 0.4 V at 5 mV s^−1^. The open-circuit voltage, galvanostatic discharge, rate discharge, and galvanostatic discharge–charge cycling (discharging for10 min, then charging for 10 min at 5 mA cm^−2^) were evaluated using a Land CT3001A multichannel battery testing system.

DFT details: All DFT calculations were performed using Quantum Espresso (QE).^[1, 2]^ The generalized gradient approximation (GGA) with the Perdew–Burke–Ernzerhof (PBE) functional was used to describe The exchange-correlation energies. ^[3,V4]^ The pseudopotentials of Fe, Pt, C, N, O, and H were generated using The projected augmented wave (PAW) method.^[5]^ To achieve a balance between the computational effort and accuracy, the energy cutoff was set to 800 Ry for the charge density and 200 Ry for the wave function to generate ground-state eigenvalues. The geometry relaxation, ensuring the energy difference of consecutive steps was less than 10^−5^ eV and the forces on each atom were less than 0.01 eV Å^−1^, was carried out through the application BFGS quasi-Newton algorithm. Monkhorst Pack grids of special points with a separation of 0.03 Å^−1^ were used for Brillouin zone sampling. To minimize the interactions between the adsorbed lithium and its periodic images, a vacuum space of 15 Å was used in the z-direction, preventing interactions with the periodic images of the layer. The DFT-D3 approach was used to consider Van der Waals interactions.^[6, 7]^ DFT + U approach^[8, 9]^ was adopted to treat the localized 3d electrons of Fe and Pt.^[10]^ The projwfc.x and epsilon.x programs in the QE package were used to obtain the electron DOS and dielectric function.

1. **Figures**

**
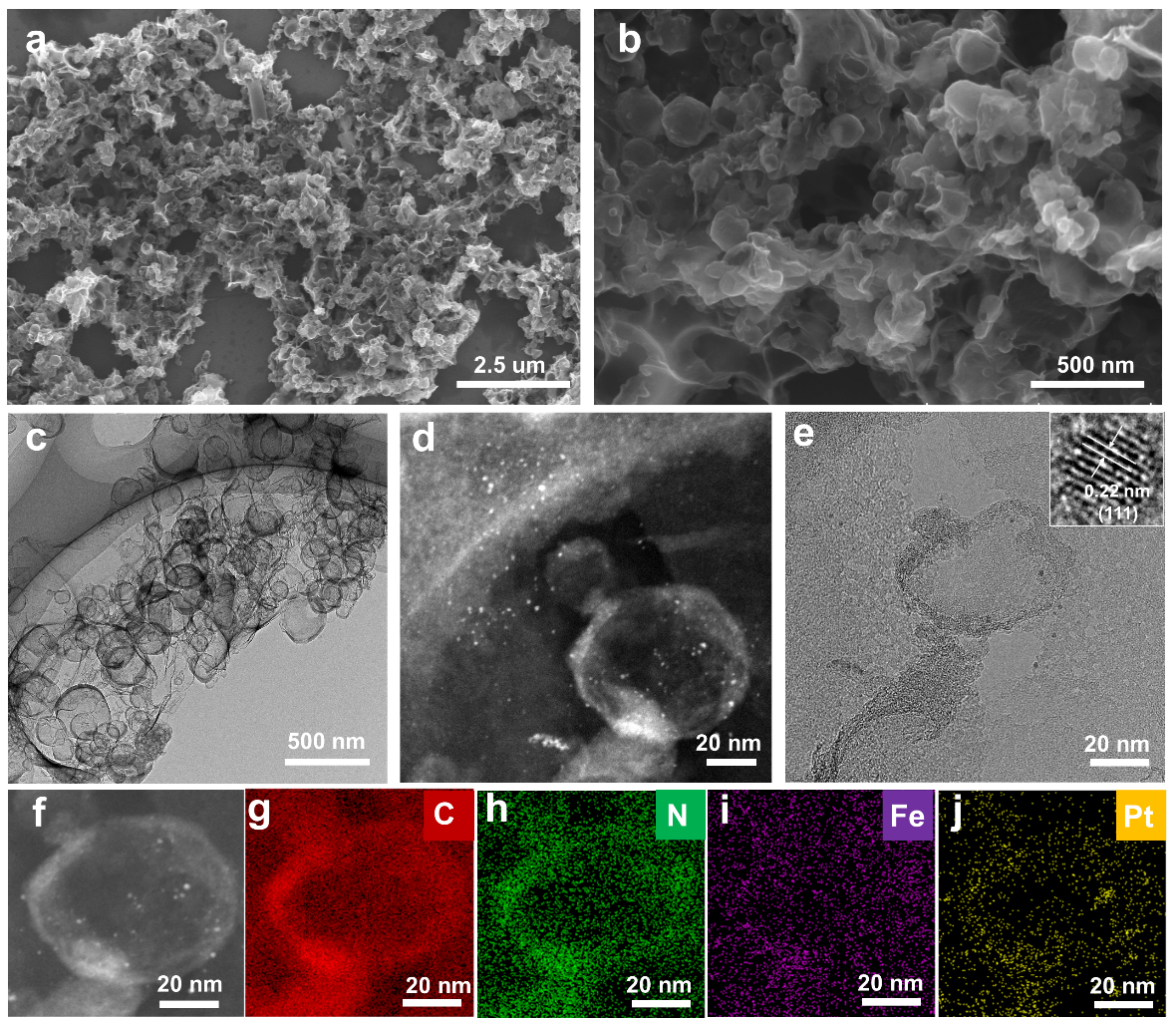
**

**Figure S1.** Electron microscopy characterization of PtFe_NPs_@PtFe_SAs_-N-C. (a-b) SEM images; (c) Low magnification TEM image; (d-e) High magnification TEM images. Inset in Figure c is the enlarged HRTEM image; (f-j) STEM image and EDS elemental mappings of C, N, Fe, Pt.


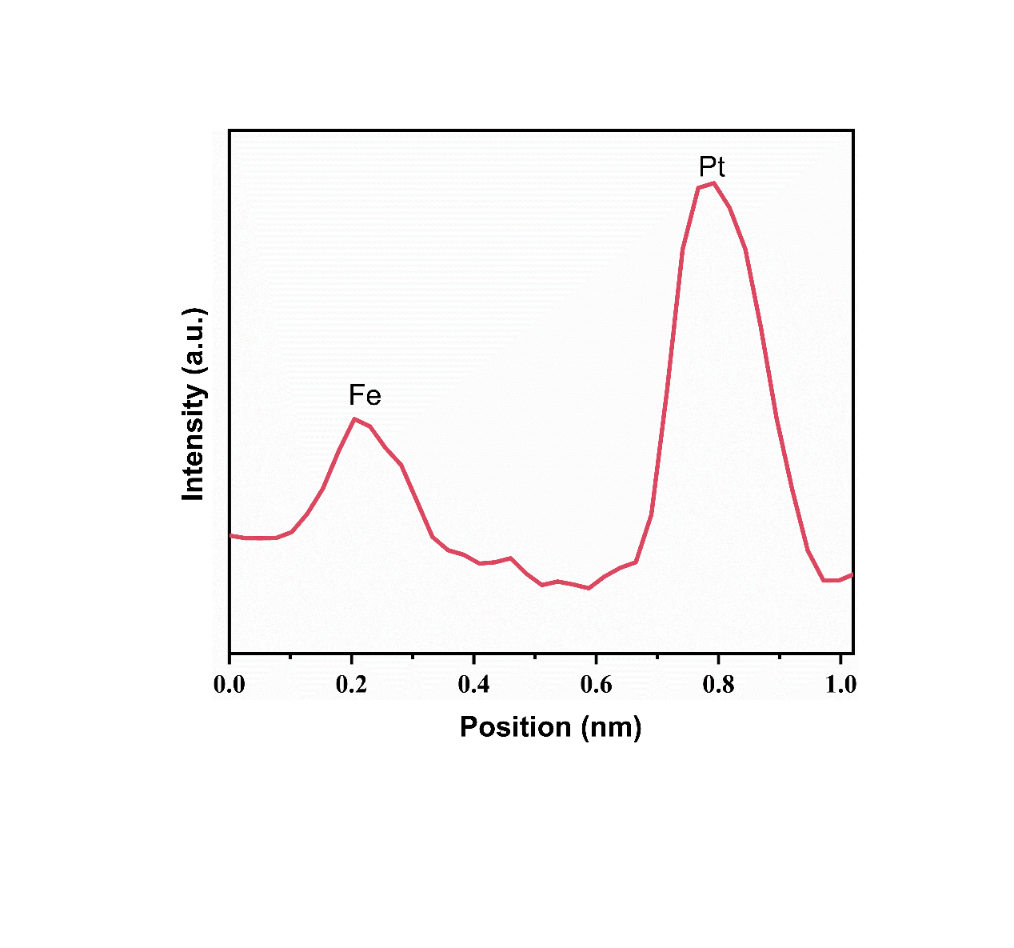


**Figure S2.** Intensity profiles of Fe and Pt atoms for PtFe_NPs_@PtFe_SAs_-N-C.


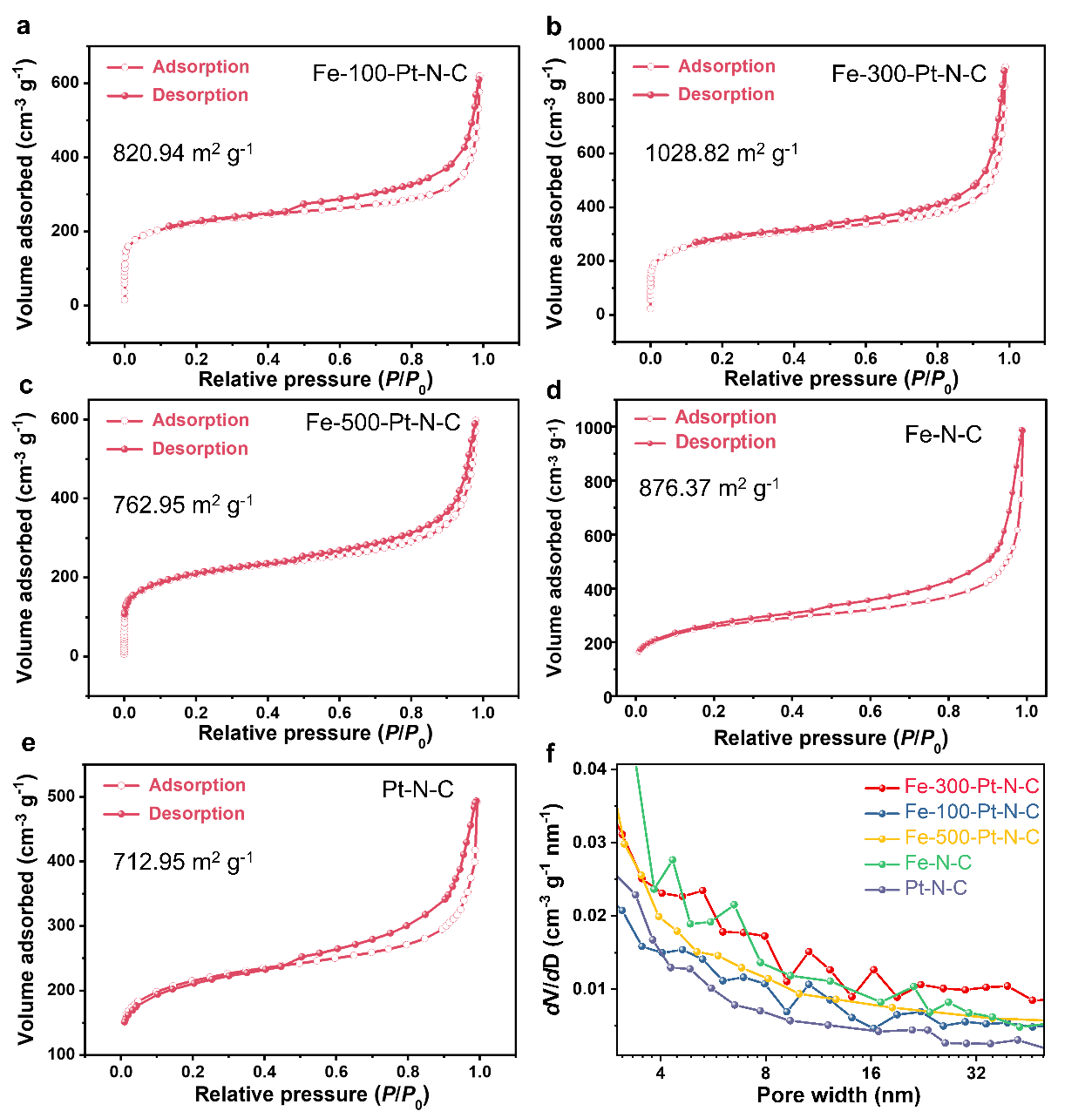


**Figure S3.** N_2_ adsorption-desorption isotherms of (a) Fe-100-Pt-N-C, (b) Fe-300-Pt-N-C, (c) Fe-500-Pt-N-C, (d) Fe-N-C; (e) Pt-N-C; (f) their corresponding pore size distributions.

**
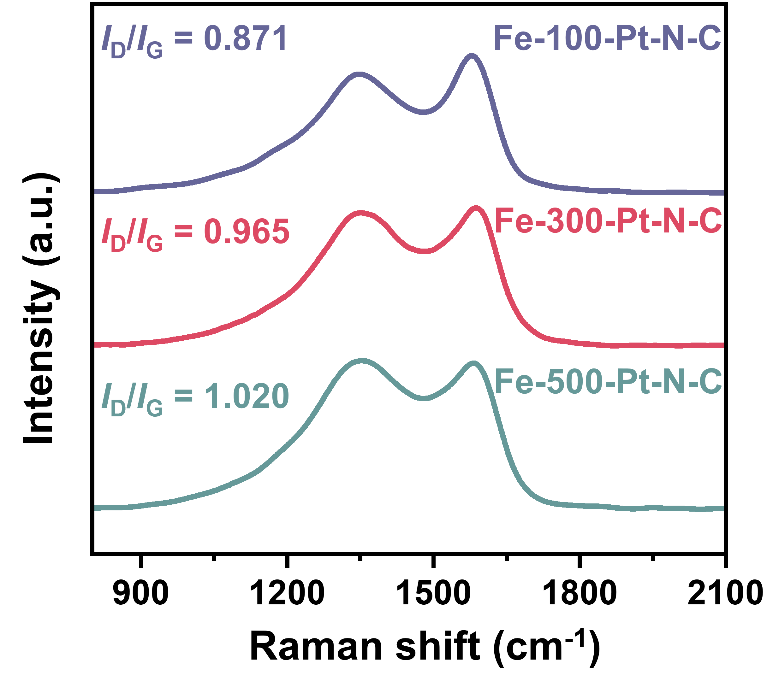
**

**Figure S4.** Raman spectra of Fe-100-Pt-N-C, Fe-300-Pt-N-C, Fe-500-Pt-N-C.


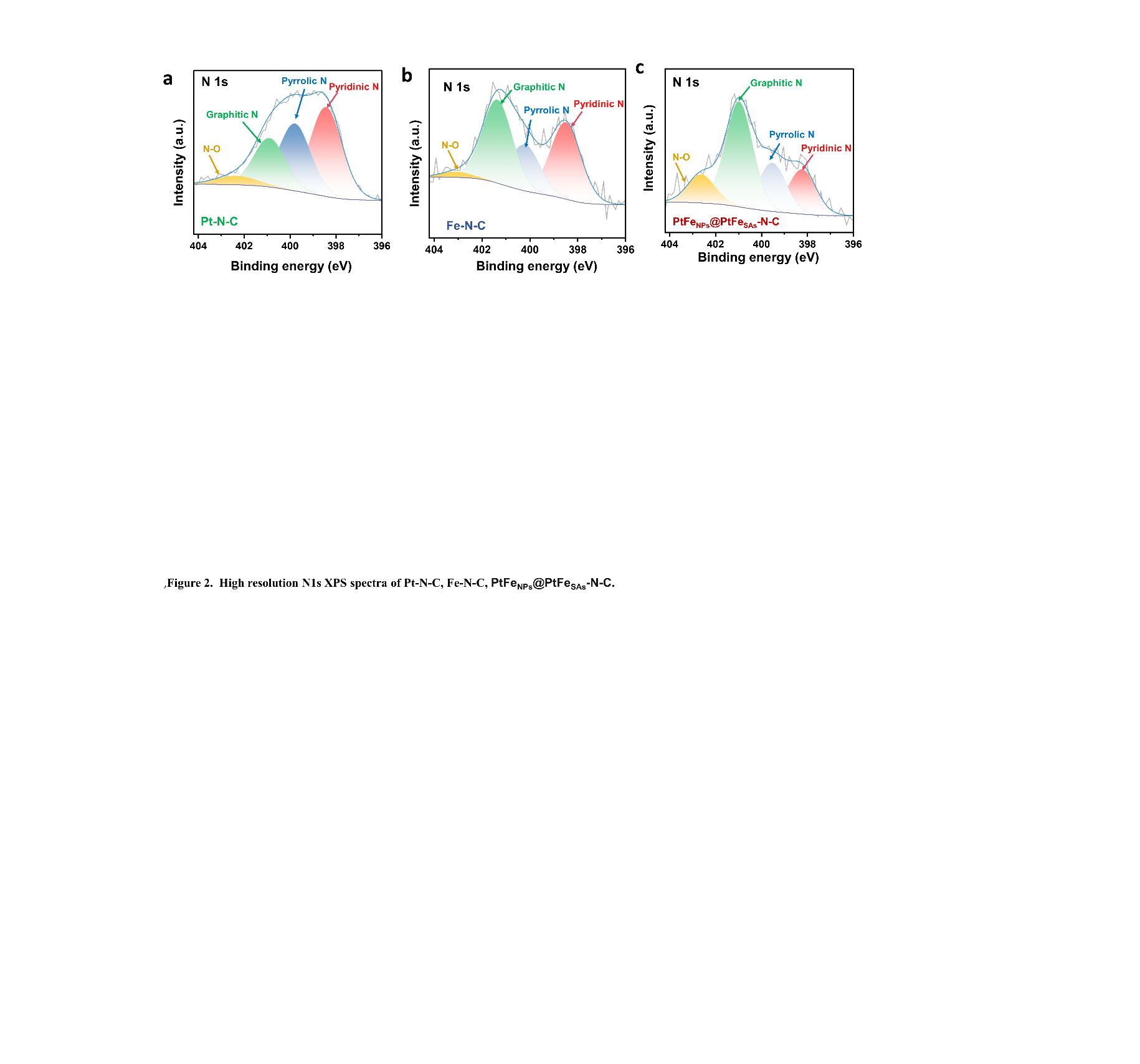


**Figure S5.** High resolution N 1s XPS spectra of (a) Pt-N-C, (b) Fe-N-C, (c) PtFe_NPs_@PtFe_SAs_-N-C.


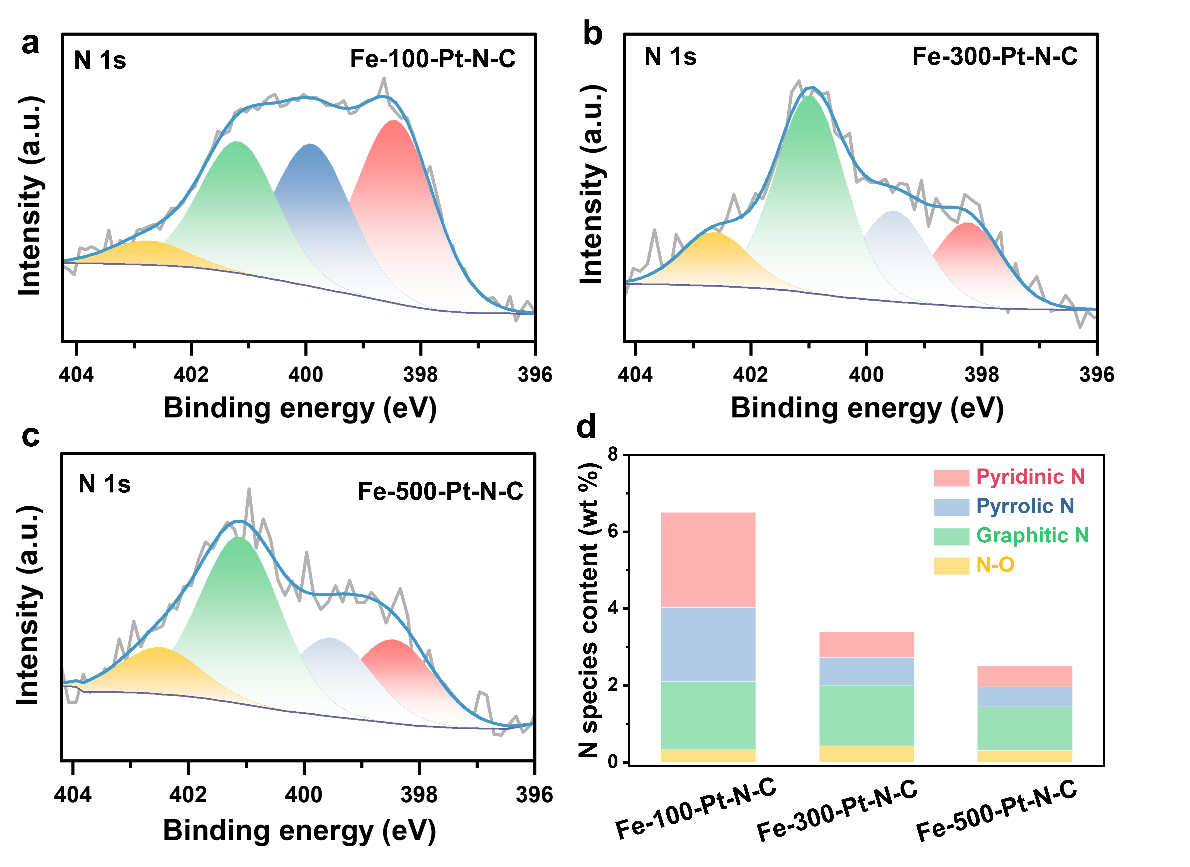


**Figure S6.** High-resolution N 1s XPS spectra of (a) Fe-100-Pt-N-C, (b) Fe-300-Pt-N-C, (c) Fe-500-Pt-N-C; (d) The N species contents distributions.

By varying the amount of FeCl_2_ addition, we further demonstrated the role of Fe species in the decrease in edge-N (pyrrolic N and pyridinic N) doping, the content of edge-N in Fe-100-Pt-N-C, Fe-300-Pt-N-C, and Fe-500-Pt-N-C is 4.39 wt%, 1.4 wt%, and 1.06 wt%, respectively, while the content of graphic N remains relatively stable for Fe-100-Pt-N-C (1.7 wt%), and Fe-300-Pt-N-C (1.57 wt%). However, it decreases sharply for Fe-500-Pt-N-C (1.13 wt%) due to the excess Fe addition.

**Figure S7.** WT-EXAFS plots of (a) Pt foil; (b) PtO_2_; (c) PtFe_NPs_@PtFe_SAs_-N-C.

**Figure S8.** WT-EXAFS plots of (a) Fe foil; (b) FeO; (c) Fe_2_O_3_; (d) PtFe_NPs_@PtFe_SAs_-N-C.


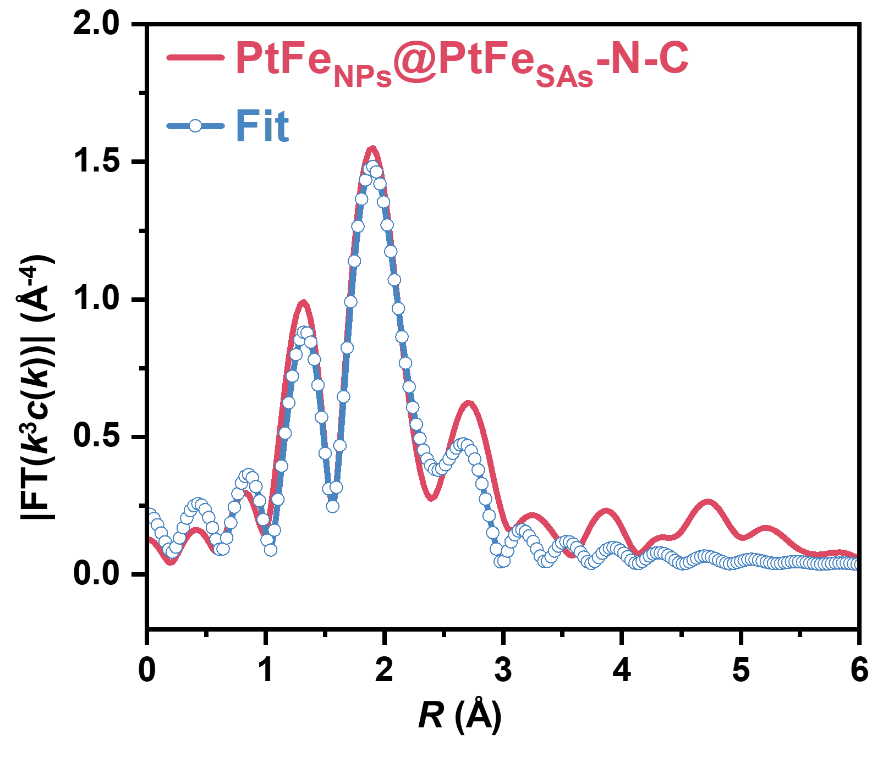


**Figure S9.** EXAFS fitting curves of PtFe_NPs_@PtFe_SAs_-N-C in the R-space of the Pt L_3_-edge.


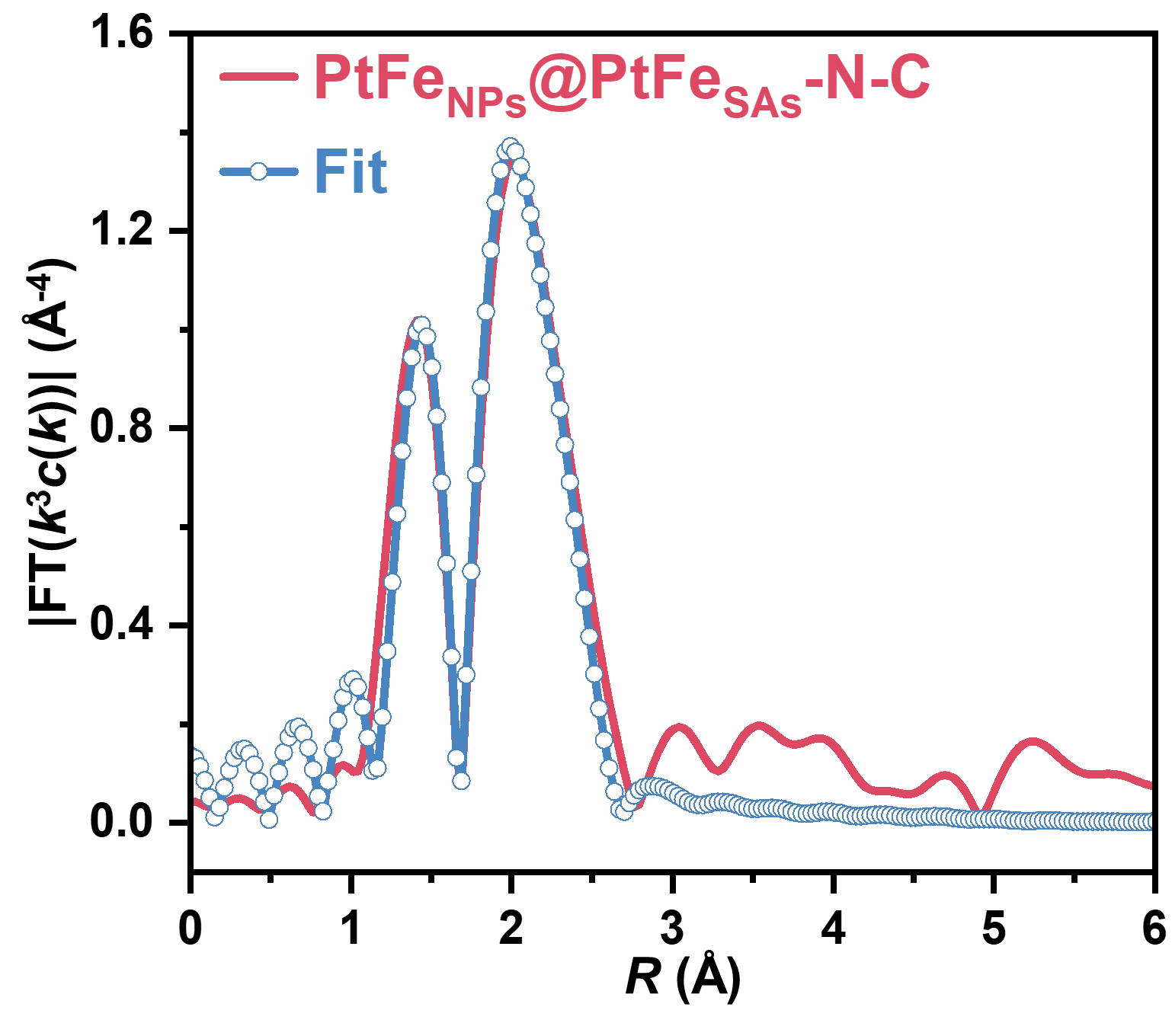


**Figure S10.** EXAFS fitting curves of PtFe_NPs_@PtFe_SAs_-N-C in the R-space of the Fe K-edge.


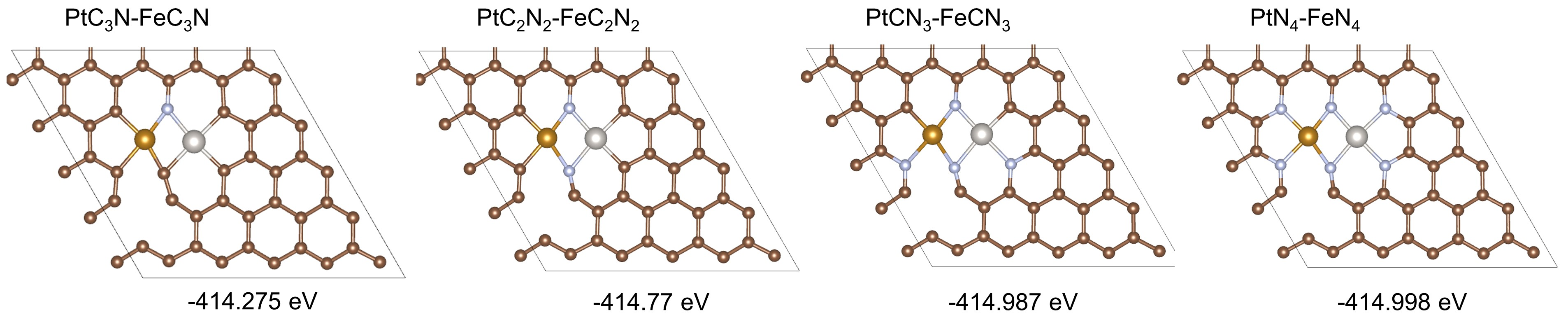


**Figure S11**. Calculated formation energies for the possible Fe, Pt dual atom-bonding configurations at the edged defect of graphene.


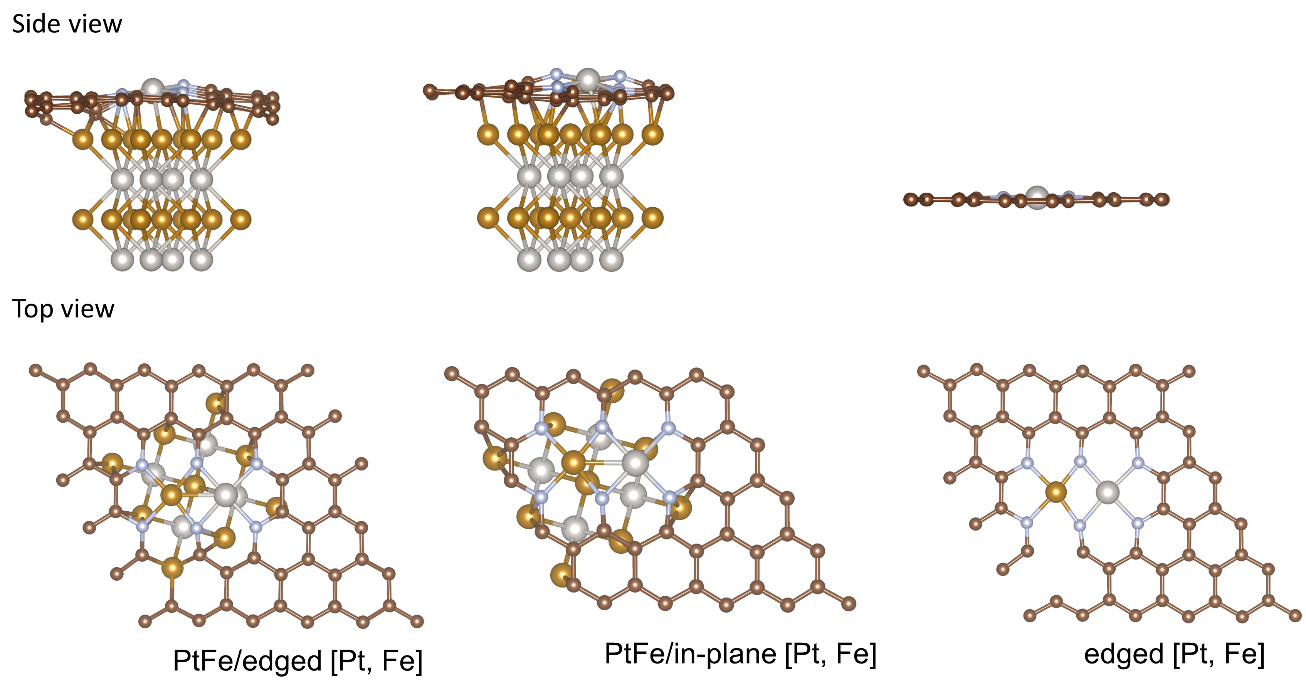


**Figure S12.** Surface structures of PtFe/edged [Pt, Fe], PtFe/in-plane [Pt, Fe], edged [Pt, Fe].


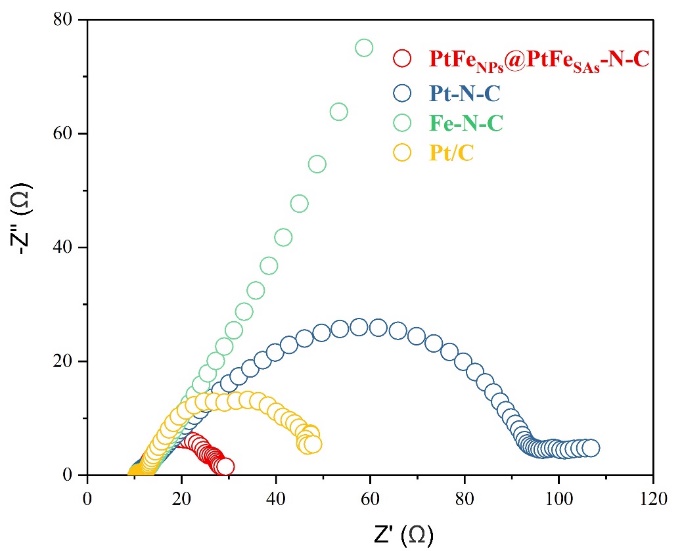


**Figure S13.** EIS Nyquist plots for the HER of the set of samples in 0.5 M H_2_SO_4_.


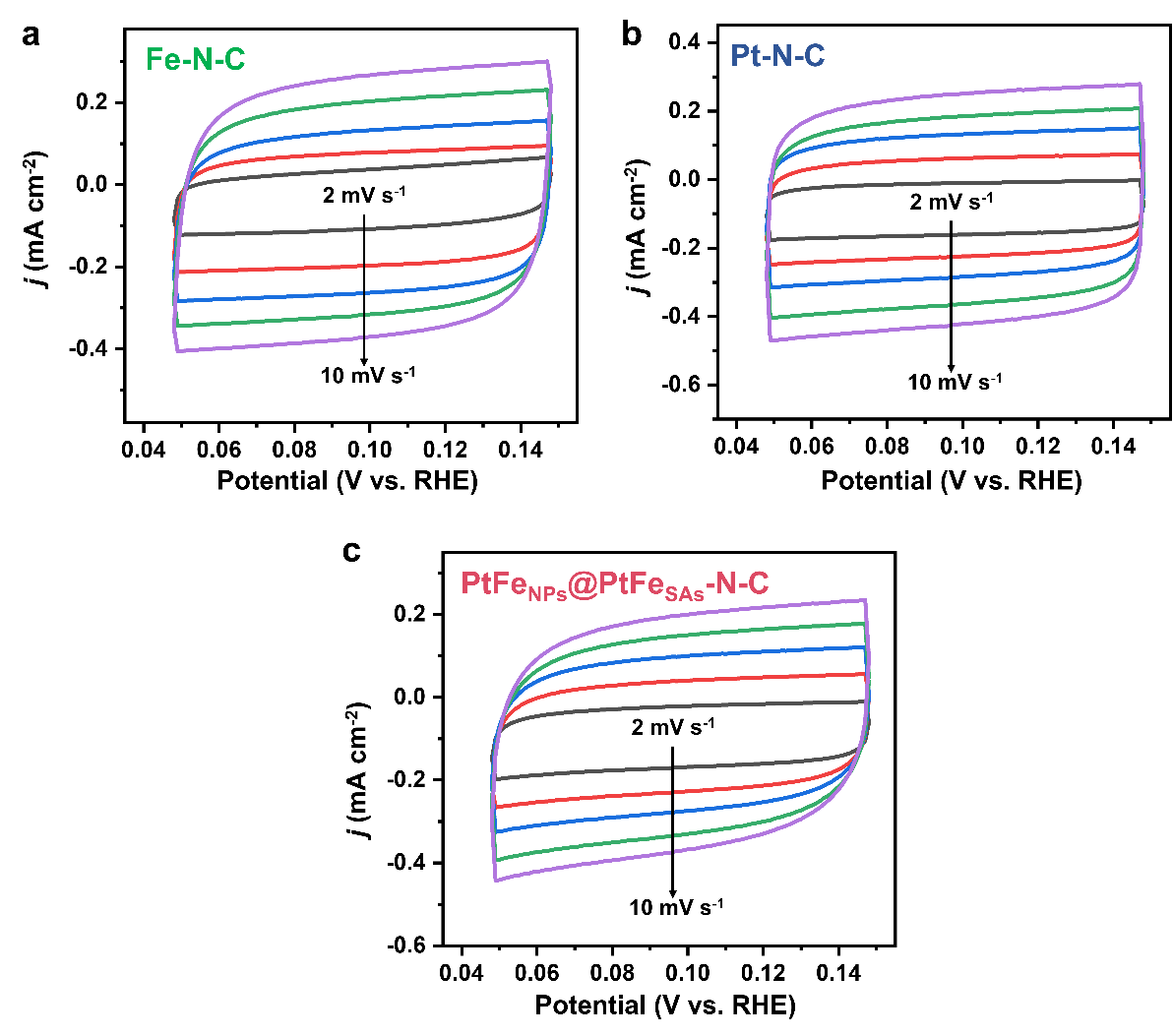


**Figure S14.** CV curves measured from 2 to 10 mV s^-1^ of (a) Fe-N-C, (b) Pt-N-C, (c) PtFe_NPs_@PtFe_SAs_-N-C in 0.5 M H_2_SO_4_ for HER; (d) Plot of the current density vs. the scan rate of Fe-N-C, Pt-N-C, and PtFe_NPs_@PtFe_SAs_-N-C.

**
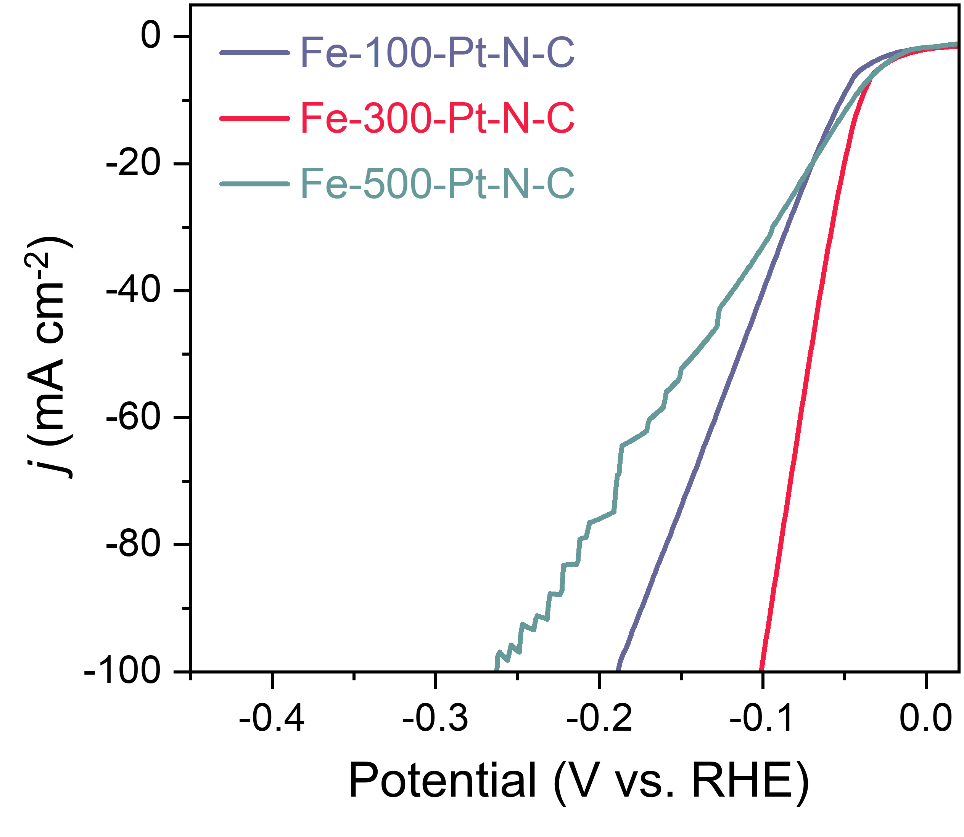
**

**Figure S15.** LSV curves of Fe-100-Pt-N-C, Fe-300-Pt-N-C, Fe-500-Pt-N-C for HER in 0.5 M H_2_SO_4_.


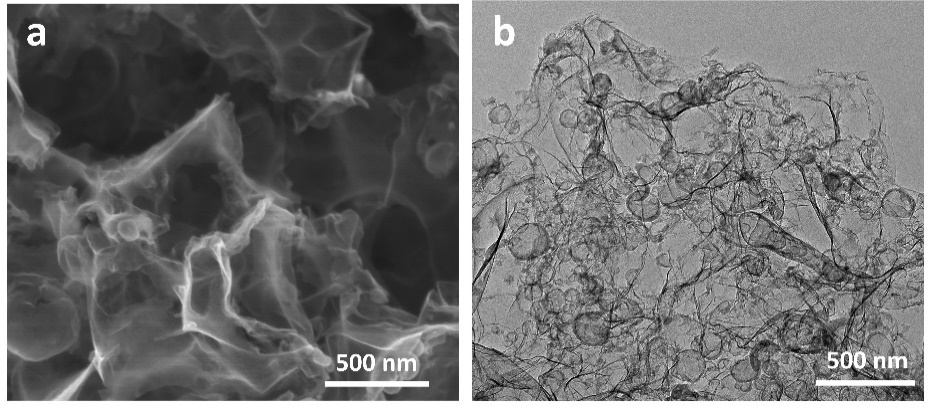


**Figure S16.** (a) SEM image; (b) TEM images of the used PtFe_NPs_@PtFe_SAs_-N-C after durability test for HER in acid media.


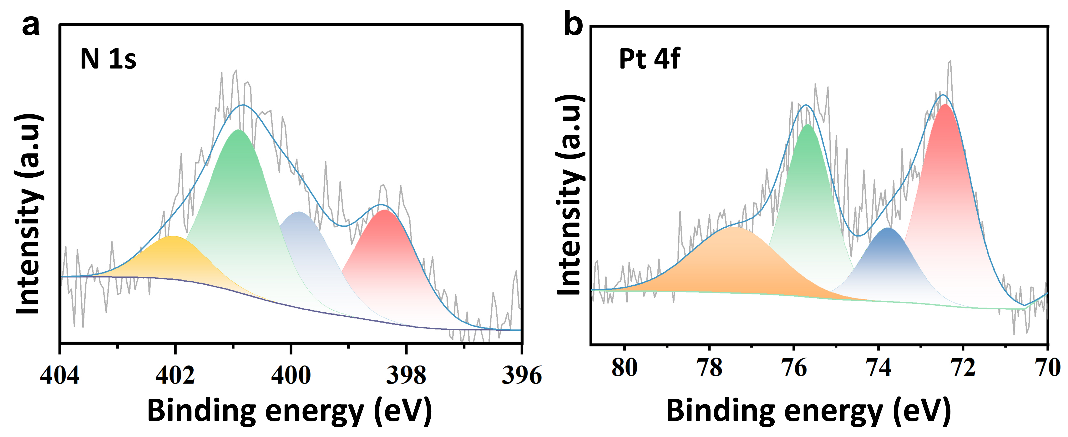


**Figure S17.** High resolution (a) N 1s, (b) Pt 4f XPS spectra of the used PtFe_NPs_@PtFe_SAs_-N-C after durability test for HER in acid media.

**
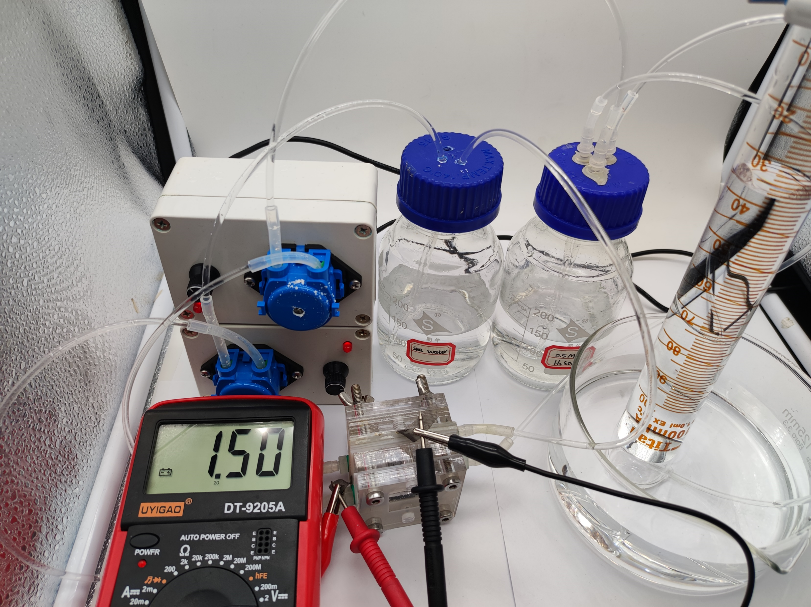
**

**Figure S18.** Digital photograph of open-circuit voltage for the flow seawater-Al/acid hybrid fuel cell.

.

**
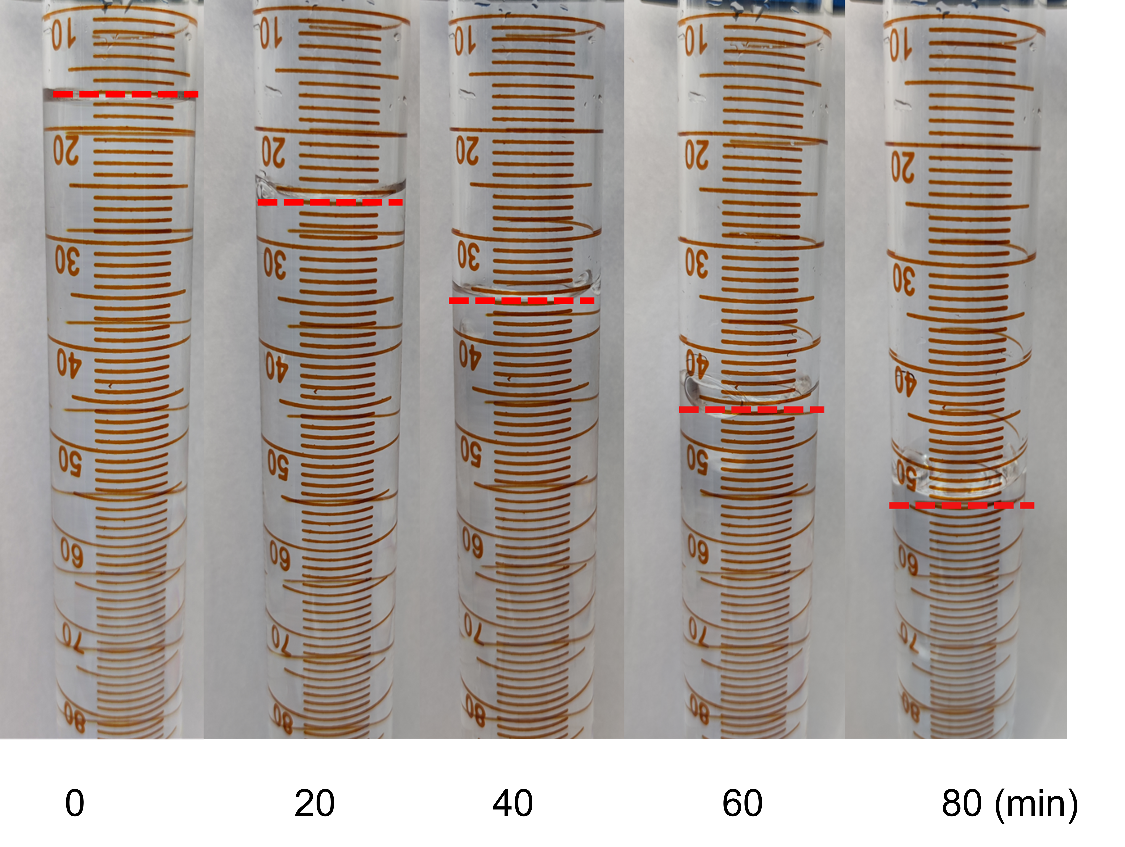
**

**Figure S19.** Photograph of the hydrogen gas collection tubes at different time connected with flow seawater-Al/acid hybrid fuel cell.

**

**Figure S20.** CV curves of PtFe_NPs_@PtFe_SAs_-N-C in N_2_ and O_2_-saturated 0.1 M KOH.

The cyclic voltammetry (CV) curves (Figure S17) of PtFe_NPs_@PtFe_SAs_-N-C in the O_2_-staturated electrolyte show a prominent cathodic peak, which is absent in the N_2_-saturated electrolyte, confirming that PtFe_NPs_@PtFe_SAs_-N-C can drive the ORR.

**
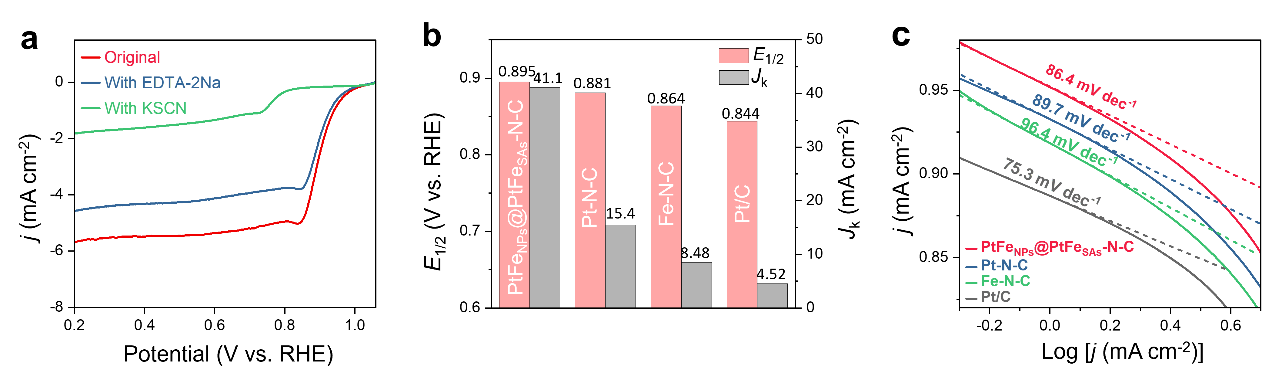
**

**Figure S21.** (a) LSV curves of PtFe_NPs_@PtFe_SAs_-N-C in 0.1 M KOH with the addition of KSCN or EDTA-2Na; (b) J_k_ at 0.85 V, and E_1/2_ of PtFe_NPs_@PtFe_SAs_-N-C, Pt-N-C, Fe-N-C and Pt/C; (c) Tafel plots of PtFe_NPs_@PtFe_SAs_-N-C, Pt-N-C, Fe-N-C and Pt/C.


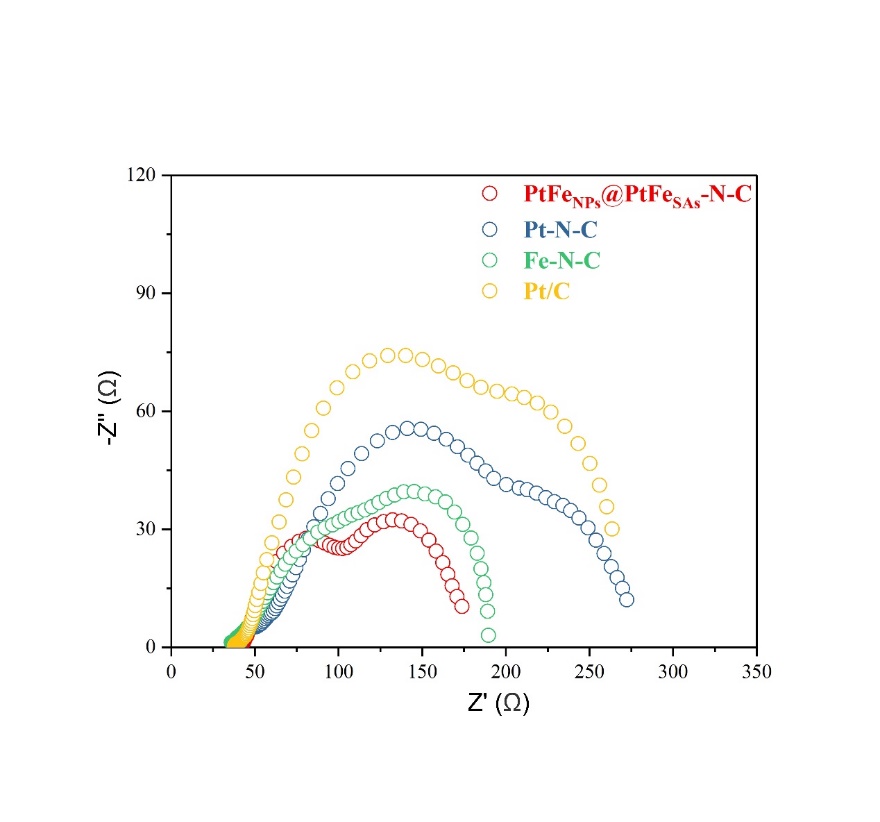


**Figure S22.** EIS Nyquist plots for the ORR of the set of samples in 0.1 MKOH.

**
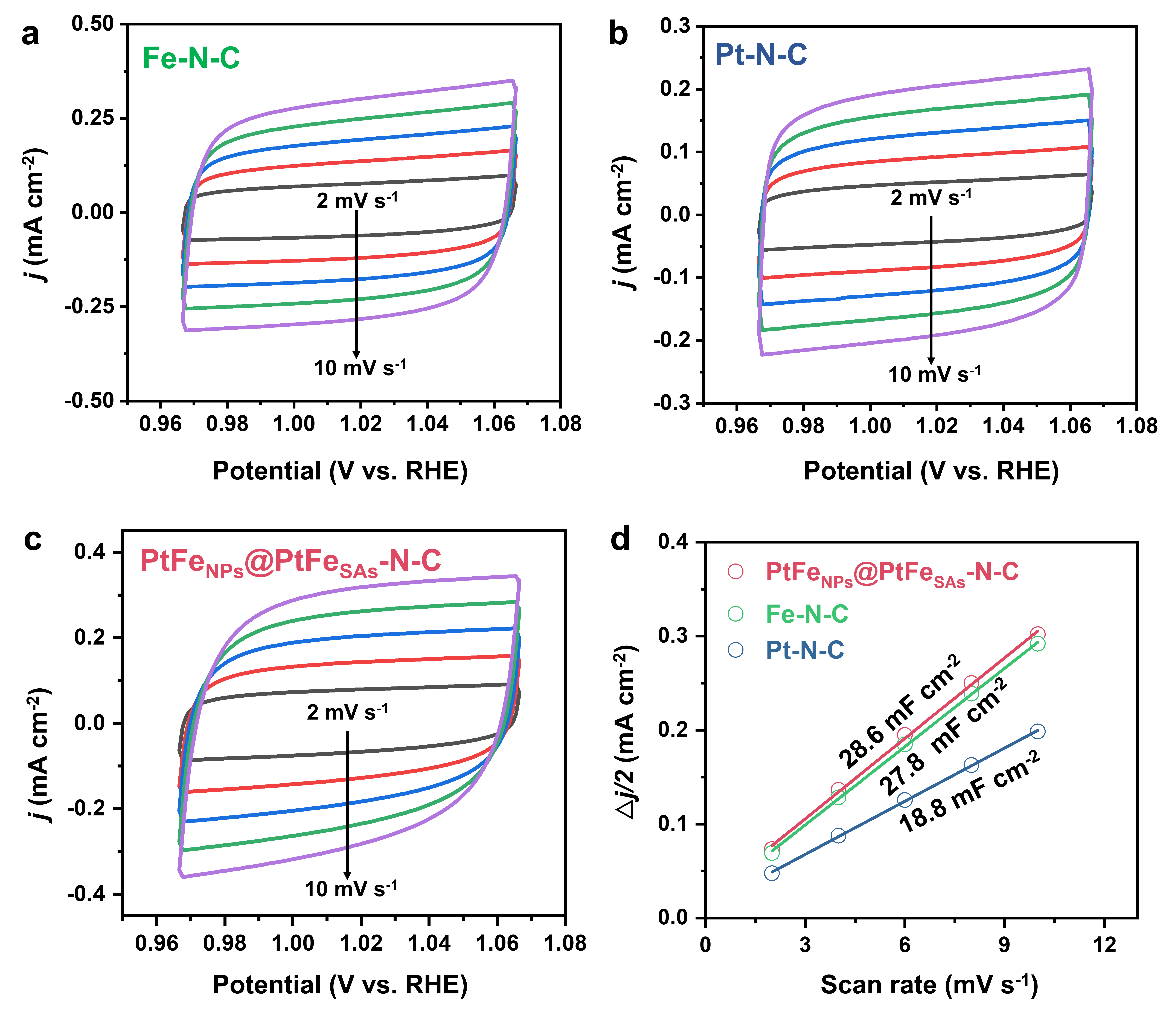
**

**Figure S23.** CV curves measured from 2 to 10 mV s^-1^ of (a) Fe-N-C, (b) Pt-N-C, (c) PtFe_NPs_@PtFe_SAs_-N-C in 0.1 M KOH for ORR; (d) Plot of the current density vs. the scan rate of Fe-N-C, Pt-N-C, and PtFe_NPs_@PtFe_SAs_-N-C.


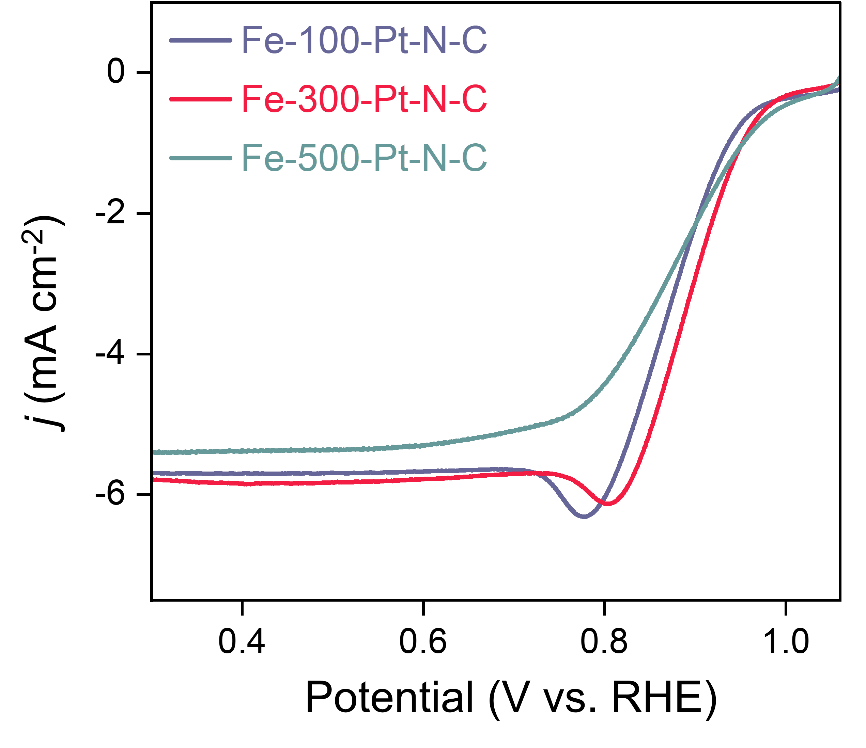


**Figure S24.** RDE polarization curves of Fe-100-Pt-N-C, Fe-300-Pt-N-C and Fe-500-Pt-N-C with a scan rate of 5 mV s^–1^ at 1600 rpm in 0.1 M KOH.


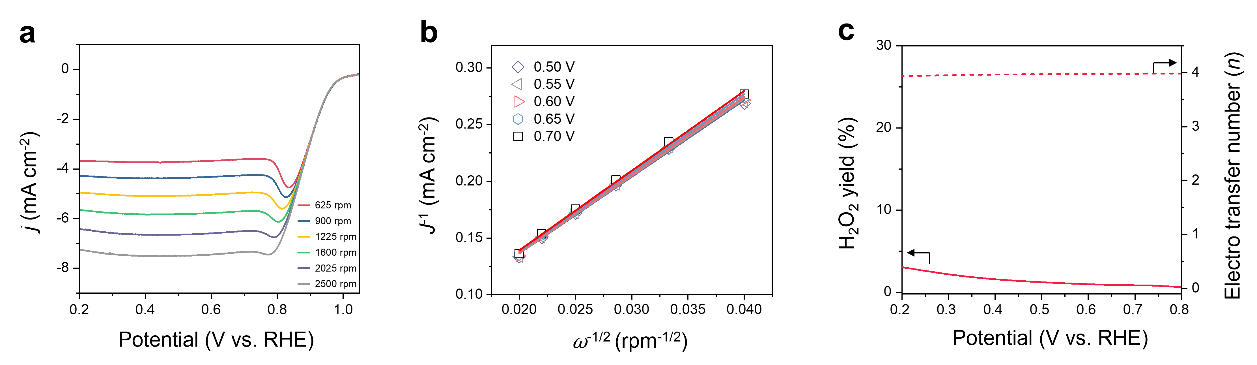


**Figure S25.** (a) Polarization curves of PtFe_NPs_@PtFe_SAs_-N-C at rotating speed from 625 to 2500 rpm; (b) the responding K-L plot; (c) Electron transfer number (top) and H_2_O_2_ yield (bottom) of PtFe_NPs_@PtFe_SAs_-N-C obtained from RRDE.


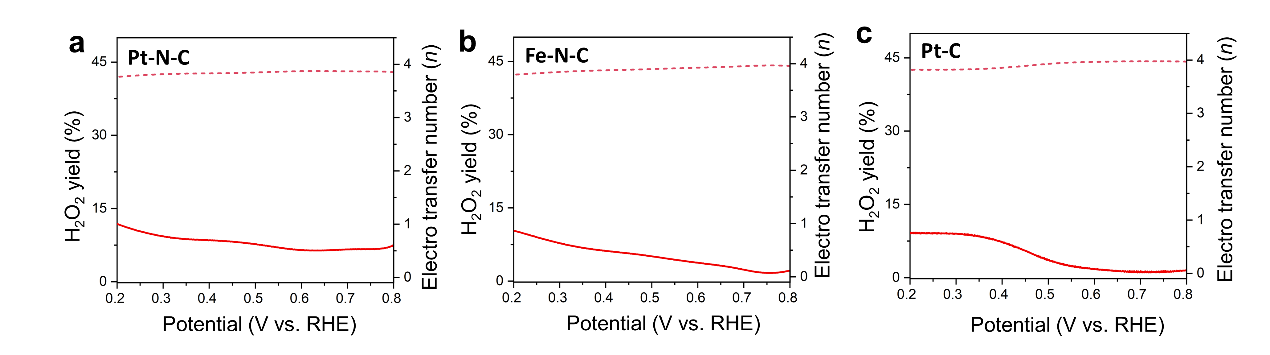


**Figure S26.** Electron transfer number (top) and H_2_O_2_ yield (bottom) of Pt-N-C (a), Fe-N-C (b), Pt-C (c) obtained from RRDE

**
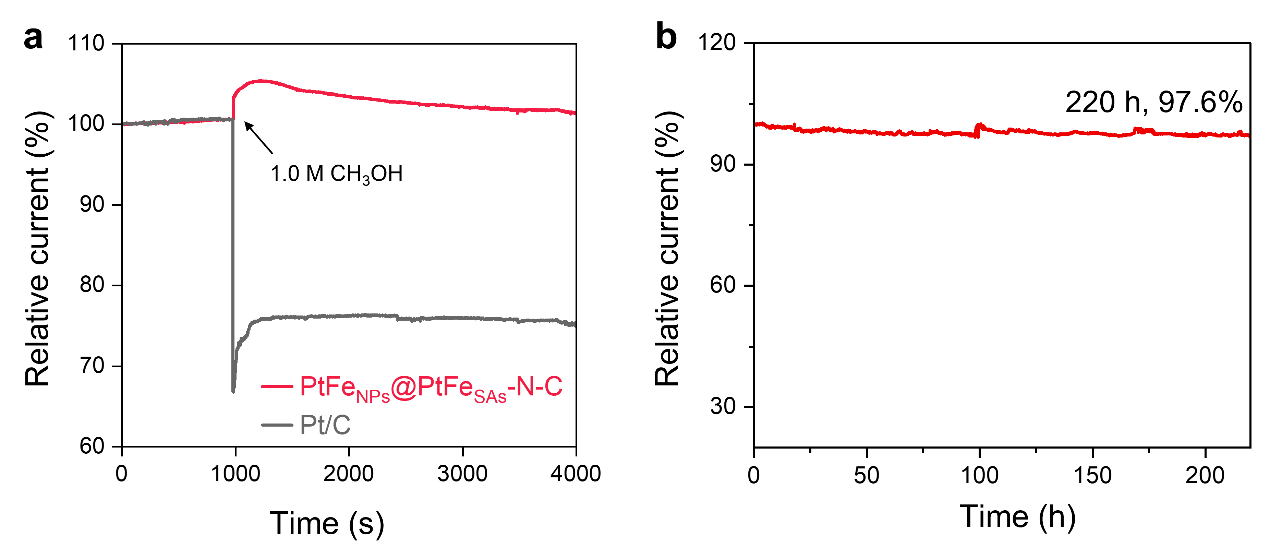
**

**Figure S27.** (a) Chronoamperometric curves of PtFe_NPs_@PtFe_SAs_-N-C and Pt/C with adding 1.0 M CH_3_OH; (b) Chronoamperometric curve PtFe_NPs_@PtFe_SAs_-N-C.


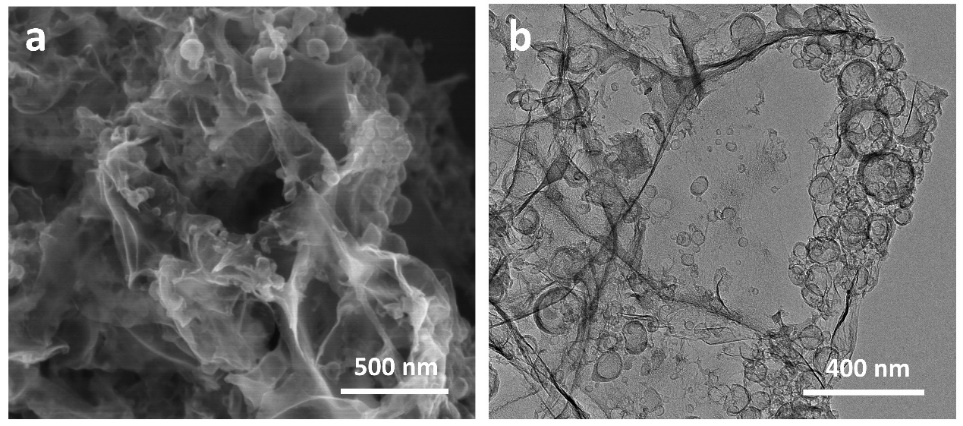


**Figure S28.** (a) SEM image; (b) TEM images of the used PtFe_NPs_@PtFe_SAs_-N-C after durability test for ORR in alkaline media.


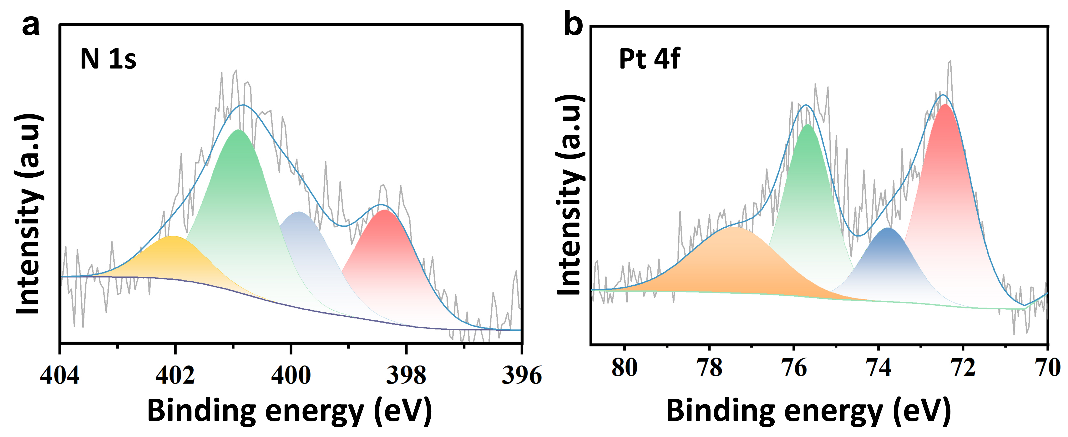


**Figure S29.** High resolution (a) N 1s, (b) Pt 4f XPS spectra of the used PtFe_NPs_@PtFe_SAs_-N-C after durability test for ORR in alkaline media.


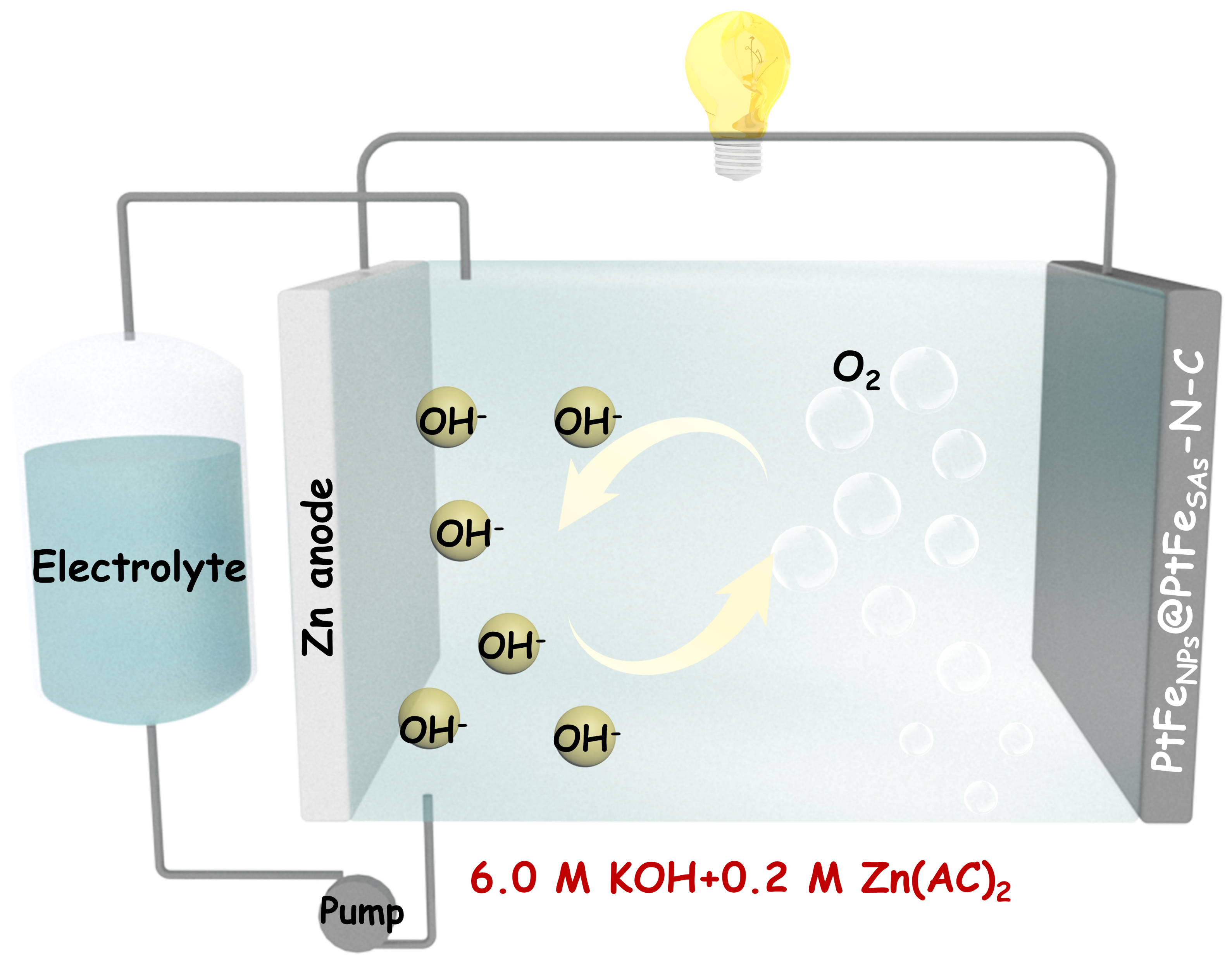


**Figure S30.** Schematic representation of flow Zn-air battery.


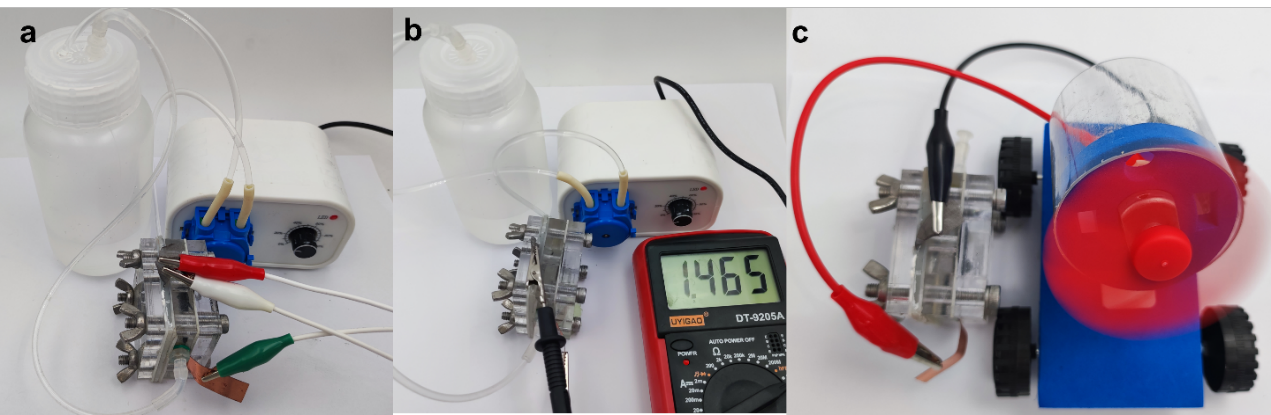


**Figure S31.** Digital photograph of PtFe_NPs_@PtFe_SAs_-N-C based flow Zn-air battery. (a) The home-made setup; (b) The open-circuit voltage for the flow Zn-air battery; (c) A miniature windmill powered by flow Zn-air battery.


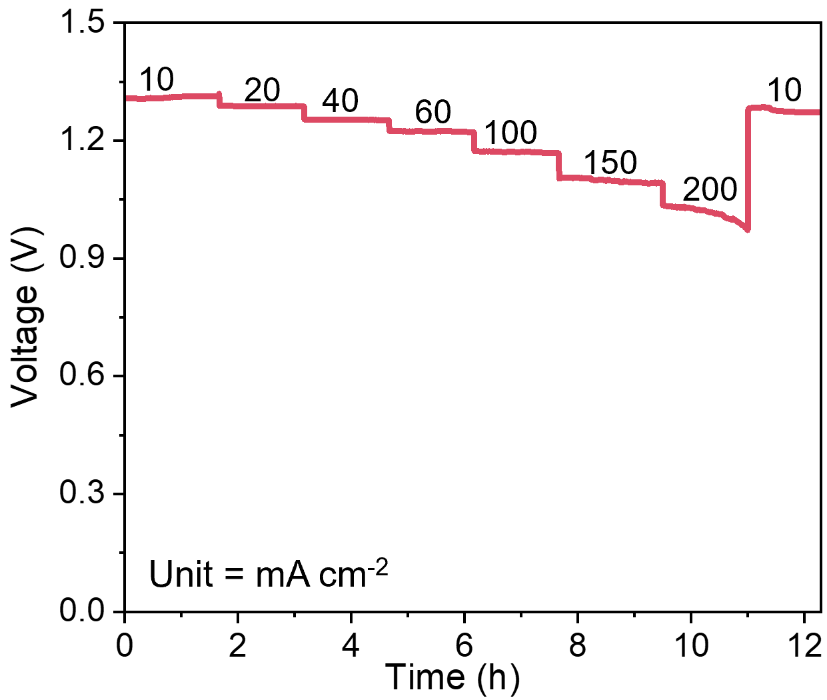


**Figure S32.** Chronopotentiometric response of PtFe_NPs_@PtFe_SAs_-N-C ZAB at different current densities.


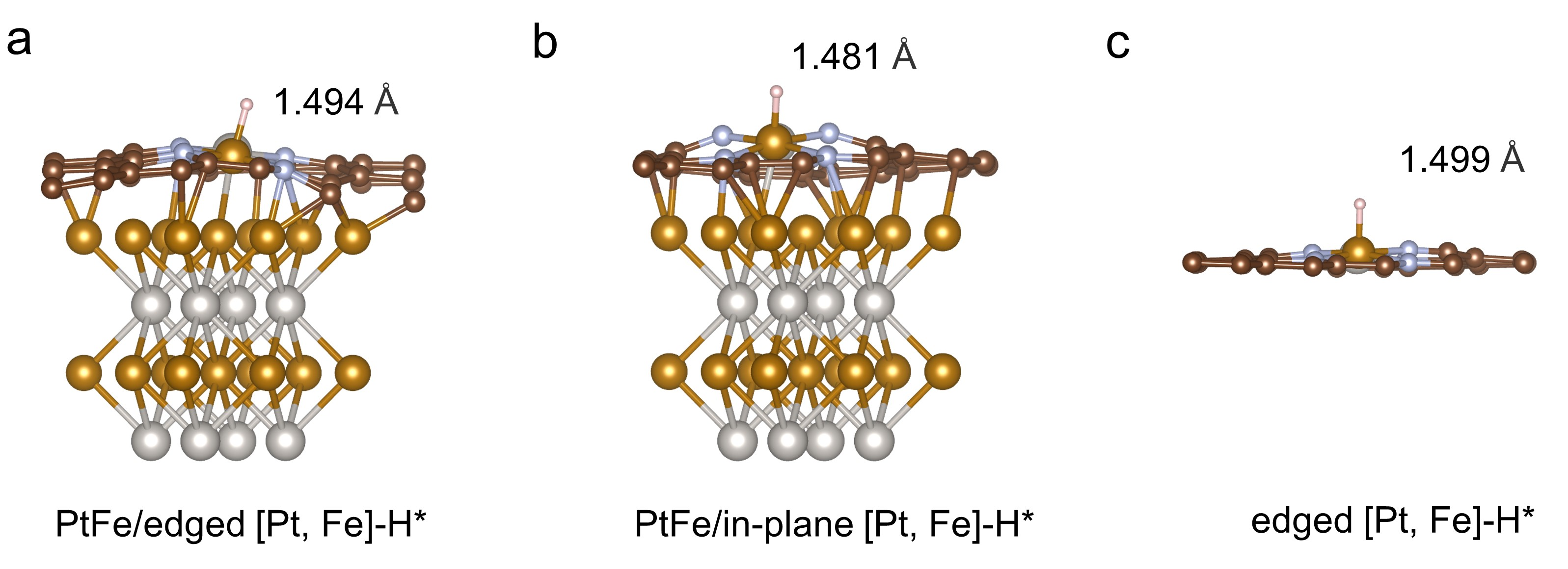


**Figure S33.** The Fe-H bond length of the (a) PtFe/edged [Pt, Fe], (b) PtFe/in-plane [Pt, Fe], (c) edged [Pt, Fe] structure.


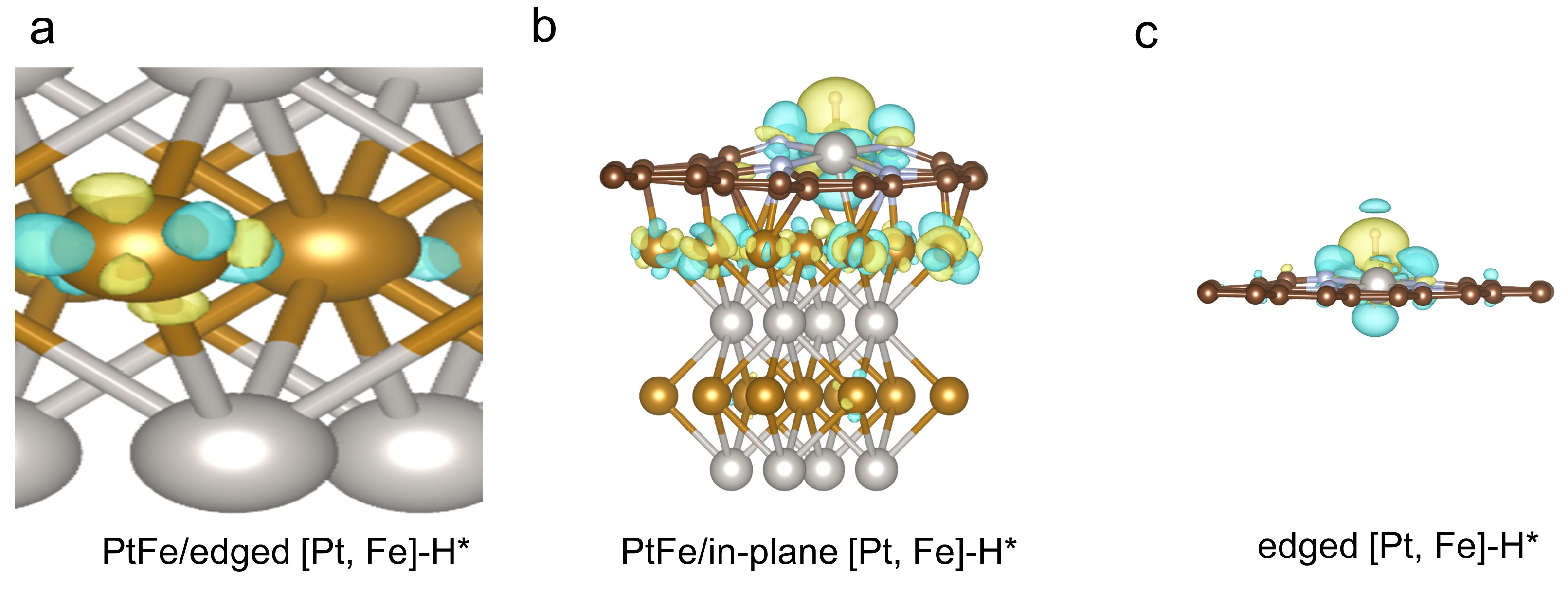


**Figure S34.** The charge density difference of the H* adsorbed on the (a) PtFe/edged [Pt, Fe], (b) PtFe/in-plane [Pt, Fe], (c) edged [Pt, Fe].


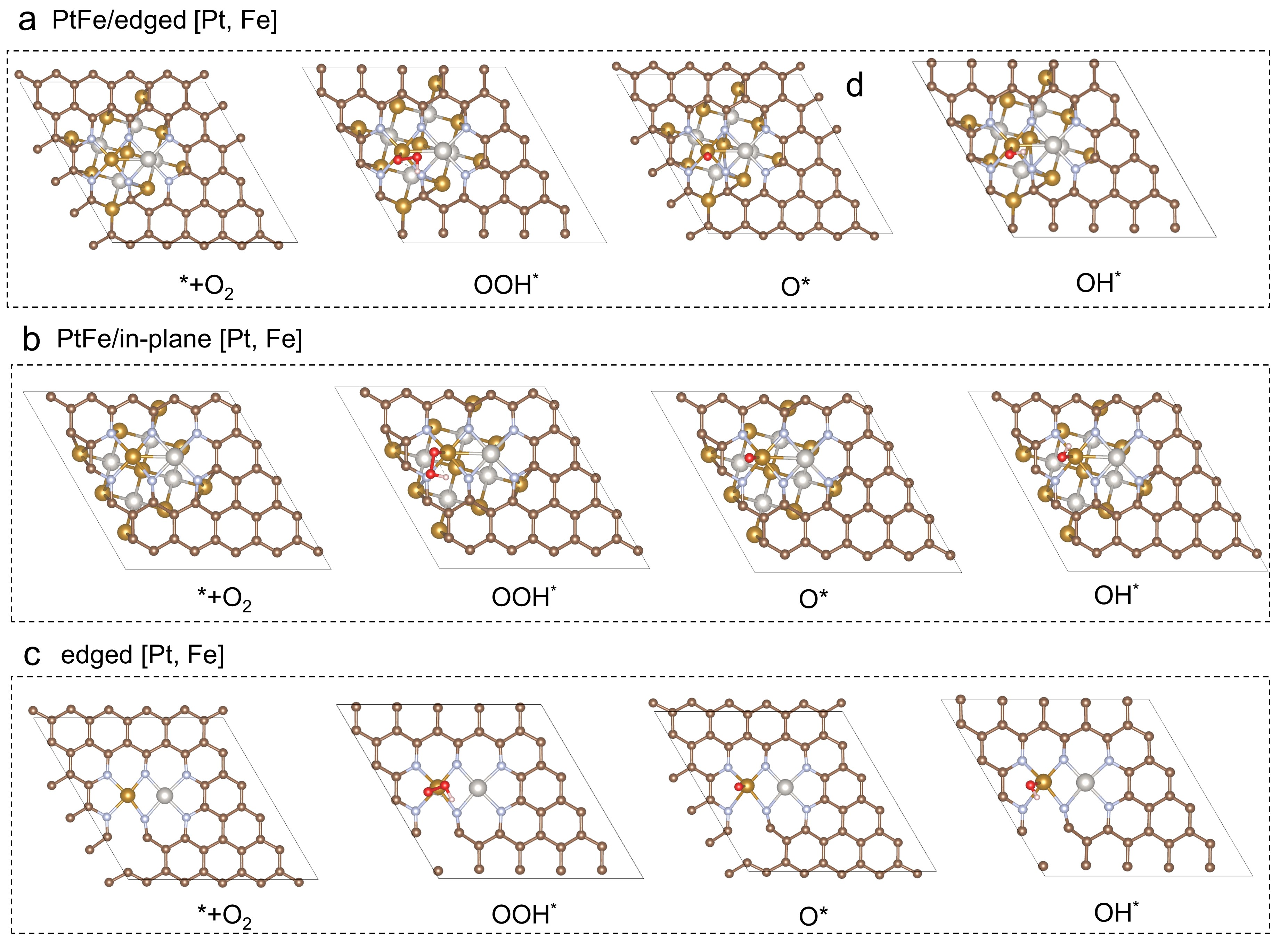


**Figure S35.** Optimized intermediate structures of the (a) PtFe/edged [Pt, Fe], (b) PtFe/in-plane [Pt, Fe], (c) edged [Pt, Fe] systems for each reaction step of the 4 e^-^ reaction pathways.


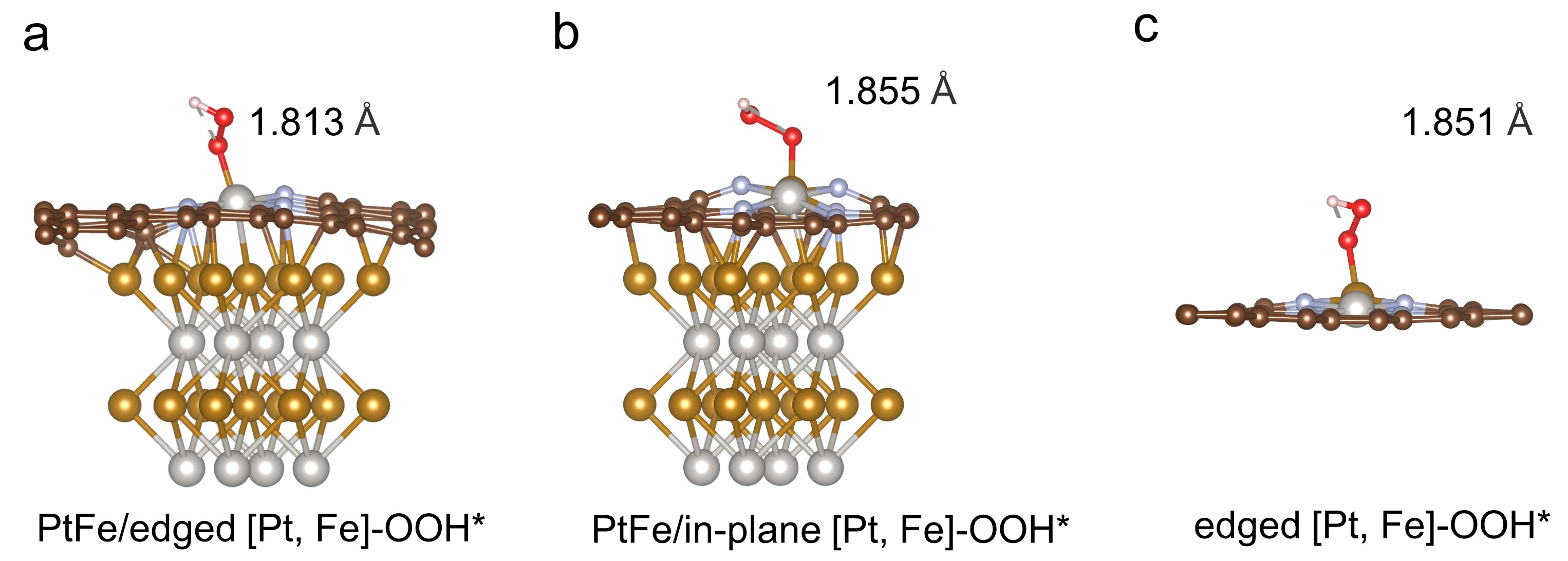


**Figure S36.** The Fe-O bond length of the (a) PtFe/edged [Pt, Fe], (b) PtFe/in-plane [Pt, Fe], (c) edged [Pt, Fe] structure for OOH* adsorption.


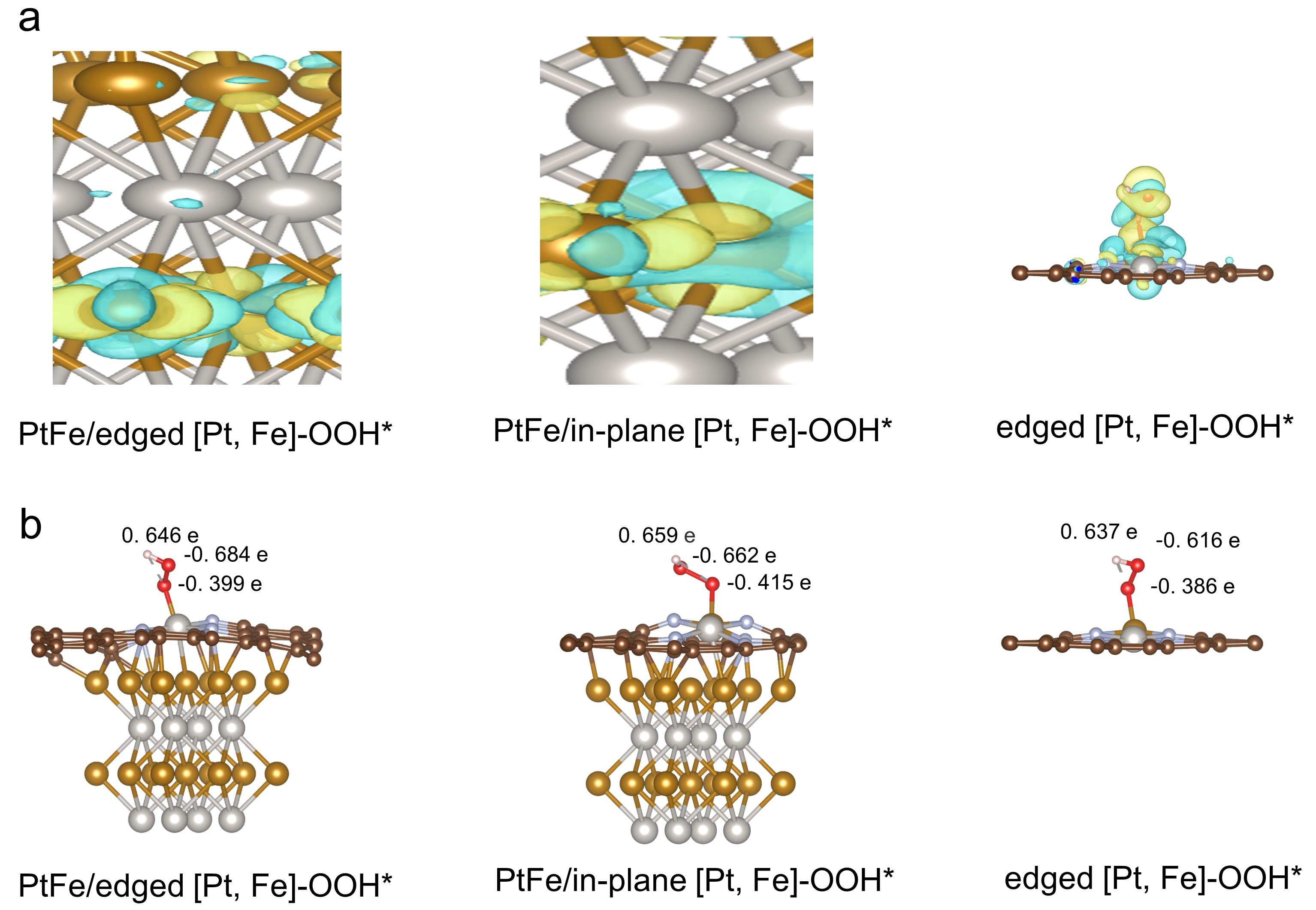


**Figure S37.** (a) The charge density difference and (b) Bader charge of the OOH* intermediate adsorbed on the PtFe/edged [Pt, Fe], PtFe/in-plane [Pt, Fe], edged [Pt, Fe].

**4. Tables**

**Table S1.** ICP-OES analysis results of PtFe_NPs_@PtFe_SAs_-N-C electrocatalyst.

| **Samples** | **Pt (wt.%)** | **Fe (wt.%)** |
| --- | --- | --- |
| PtFe_NPs_@PtFe_SAs_-N-C | 2.2 | 0.84 |

**Table S2.** EXAFS fitting parameters at the Pt L_3_-edge various samples (S_0_^2^=0.90)

| Samples | Path | C. N.^[a]^ | R (Å) ^[b]^ | σ^2^ (× 10^−3^ Å^2^) ^[c]^ | ΔE (eV) ^[d]^ | R factor^[e]^ |
| --- | --- | --- | --- | --- | --- | --- |
| Pt foil | Pt-Pt | 12* | 2.77* | 4.4 | 6.3 | 0.01 |
| PtO_2_ | Pt-O | 6* | 1.98* | 2.7 | 8.6 | 0.01 |
|  | Pt-Pt | 2* | 3.14* | 9.5 |  |  |
| PtFe_NPs_@PtFe_SAs_-N-C | Pt-C/N/O | 3.8 | 1.86 | 6.2 | -7.9 | 0.02 |
|  | Pt-Fe | 4.8 | 2.54 | 8.6 |  |  |

Note: ^a^C. N.: coordination numbers; ^b^R: bond distance; ^c^σ^2^: Debye-Waller factors; ^d^ΔE_0_: the inner potential correction. ^e^R factor: goodness of fit. *The experimental EXAFS fit by fixing C. N. as the known crystallographic value.

**Table S3.** EXAFS fitting parameters at the Fe K-edge various samples (S_0_^2^=0.89).

| Samples | Path | C. N.^[a]^ | R (Å) ^[b]^ | σ^2^ (× 10^−3^ Å^2^) ^[c]^ | | ΔE (eV) ^[d]^ | | R factor^[e]^ |
| --- | --- | --- | --- | --- | --- | --- | --- | --- |
| Fe foil | Fe-Fe | 8* | 2.48* | 6.8 | 5.1 | | | 0.01 |
|  | Fe-Fe | 6* | 2.87* | 8.8 |  |  |  |  |
| FeO | Fe-O | 6* | 2. 5* | 8.4 | –5.3 | | | 0.01 |
|  | Fe-Fe | 12* | 3.05* | 8.6 |  |  |  |  |
| Fe_2_O_3_ | Fe-O | 3* | 1.98* | 5.6 | –3.3 | | | 0.02 |
|  | Fe-O | 3* | 2.13* | 7.6 |  |  |  |  |
|  | Fe-Fe | 3* | 3.00* | 8.9 |  |  |  |  |
|  | Fe-Fe | 3* | 3.42* | 9.6 |  |  |  |  |
| PtFe_NPs_@PtFe_SAs_-N-C | Fe-C/N/O | 3.5 | 1.82 | 9.7 | –6.7 | | 0.02 | |
|  | Fe-Pt | 4.8 | 2.53 | 4.9 |  |  |  |  |

Note: ^a^C. N.: coordination numbers; ^b^R: bond distance; ^c^σ^2^: Debye-Waller factors; ^d^ΔE_0_: the inner potential correction. ^e^R factor: goodness of fit. *The experimental EXAFS fit by fixing C. N. as the known crystallographic value.

**Table S4.** Summary of some recently reported representative Pt-based HER electrocatalysts in 0.5 M H_2_SO_4_.

| **Catalysts** | **Mass loading** | **Overpotent-ials (mV)** | **Tafel slope** **(mV dec^-1^)** | **Reference** |
| --- | --- | --- | --- | --- |
| **PtFe_NPs_@PtFe_SAs_-N-C** | **18.7 μg^Pt^_._cm**^-2^ | **41** | **36.7** | **this work** |
| Ti_3_C_2_T*_x_*-N-Pt_SA_ | 0.265 mg^cat^_._cm^-2^ | 38 | 32 | Nano Lett.  2022, 22, 1398-1405 |
| Pt@Co SAs-ZIF-NC | 0.285 mg^cat^_._cm^-2^ | 27 | 19 | Nano Energy  2021, 88, 106221 |
| Pt/np-Co_0.85_Se | 2.04 mg^cat^_._cm^-2^ | 58 | 26 | Nat. Commun.  2019, 10, 1743 |
| PtN*_x_*/TiO_2_ | – | 67 | 34 | Nano Energy  2020, 73, 104739 |
| CDs/Pt-PANI | 8.1 μg^Pt^_._cm^-2^ | 30 | 41.7 | Appl. Catal. B  2019, 257, 117905 |
| 10Pt/VS_2_/CP | – | 77 | 39.46 | ACS Nano  2020, 14, 5600-5608 |
| Pt-ACs/CoNC | 1.31 μg^Pt.^cm^-2^ | 24 | 27.7 | Nat. Commun.  2022, 13, 2430 |
| Pt_3_Co@NCNT | 0.4 mg^cat^_._cm^-2^ | 42 | 27.2 | Angew. Chem. Int. Ed.  2021, 60, 19068-19073 |
| Pt@MoS_2_ | – | 55.69 | 88.43 | Nano Energy  2021, 84, 105898 |
| Pt SAs/DG | 1.0 mg^cat^_._cm^-2^ | 23 | 25 | J. Am. Chem. Soc.  2019, 141, 4505-4509 |
| Pt-AC/DG-500 | 0.1 mg^cat^_._cm^-2^ | 21 | 27.5 | J. Am. Chem. Soc.  2020, 142, 5594-5601 |
| Pt/NiRu-OH | – | 38 | 39 | Appl. Catal. B  2020, 269, 118824 |
| Pt@DG | 0.23 mg^cat^_._cm^-2^ | 30 | 53 | J. Am. Chem. Soc.  2022, 144, 2171–2178 |
| Pt_1_@Fe-N-C | 0.4 mg^cat.^cm^-2^ | 60 | 42 | Adv. Energy Mater.  2018, 8, 1701345 |
| PtSe_2_/Pt | 0.34 mg_cat_.cm^-2^ | 42 | 53 | Angew. Chem. Int. Ed.  2021, 60, 23388-23393 |
| ALD 50-Pt/G-CNFs | – | 128 | 72 | Small Methods  2022, 6, 2101470 |

**Table S5.** Summary of some recently reported representative Al/Mg-seawater batteries.

| Electrolyte | Cathode  catalysts | Peak power density  (mW cm^-2^) | Reference |
| --- | --- | --- | --- |
| **Al-Simulated**  **seawater** | **PtFe_NPs_@PtFe_SAs_-N-C** | **109.5** | **this work** |
| Mg-Simulated  seawater | 1T/2H-MoSe_2_ | 7.69 | ACS Appl. Mater. Interfaces  2022, 14, 10246-10256 |
| Mg-Simulated  seawater | Ni@CNTs-Mo_x_C/Ni_2_P | 7.48 | Nano Energy  2023, 111, 108440 |
| Mg-Simulated  seawater | Pt-NF | 10.5 | Int. J. Hydrogen. Energy  2017, 42, 23045-23053 |
| Mg-Simulated  seawater | Ru/PEI-XC | 18.9 | J. Mater. Chem. A  2021, 9, 22934-22942 |
| Mg-Simulated  seawater | MoNi/NiMoO_4_ | 21.08 | Nano Energy  2022, 98, 107295 |
| Mg-Simulated  seawater | CoP/Co_2_P | 6.28 | J. Power Sources  2021, 486, 229351 |
| Mg-Simulated  seawater | Ni-MoO_2_ | 6.54 | ACS Sustain. Chem. Eng.  2021, 9, 13106-13113 |
| Mg-Natural  seawater | Monolith-800 | 6.14 | Matter  2020, 3, 879-891 |
| Mg-Natural  seawater | Ni/V_2_O_3_ | 17.81 | Chem. Eng. J.  2022, 450, 138079 |
| Mg-Simulated  seawater | Co_6_W_6_C-2-600 | 9.1 | Small  2022, 18, 2204443 |
| Al-Natural  seawater | NiHCF | 52 | J. Mater. Chem. A  2021, 9, 8685-8691 |

**Table S6.** Summary of some recently reported representative ORR electrocatalysts in 0.1 M KOH.

| **Catalysts** | **Mass loading** | **Half wave potentials (V)** | **Reference** |
| --- | --- | --- | --- |
| **PtFe_NPs_@PtFe_SAs_-N-C** | **18.7** **μg_Pt._cm^-2^** | **0.895** | **this work** |
| Pt_3.5%_Ni PF | 15  μg_Pt._cm^-2^ | 0.800 | Angew. Chem.  2019, 58, 15848-15854 |
| Ru-SAS/SNC | – | 0.861 | J. Am. Chem. Soc.  2022, 144, 2197-2207 |
| Pt@C_4_N | – | 0.861 | Chem. Eng. J.  2021, 426, 131347 |
| Pt_1_-N/BP | 1.56  μg_Pt._cm^-2^ | 0.870 | Nat Commun  2017, 8, 15938 |
| Pt NPC | 0.25  mg_cat._cm^-2^ | 0.850 | Angew. Chem  2021, 60, 21911-21917 |
| Co@Pd-Pt/CNT | 0.0254  mg_cat._cm^-2^ | 0.880 | Nat Commun  2019, 10, 440 |
| PtCo-NC | - | 0.850 | Nano Energy  2022, 98, 107341 |
| SA-PtCoF | 0.42  mg_cat._cm^-2^ | 0.880 | Nano Energy  2020, 71, 104597 |
| PtFeNC | 0.255  mg_cat._cm^-2^ | 0.895 | Appl Catal. B  2021, 286, 119891 |
| Fe-SASC | – | 0.870 | J. Mater. Chem. A  2021, 9, 7137-7142 |
| Fe_1_-HNC-500-850 | – | 0.842 | Adv. Mater.  2020, 32, 1906905 |
| Fe/Meso-NC-1000 | – | 0.885 | Adv. Mater.  2022, 34, 2107291 |
| SA-Fe-NC | – | 0.880 | Chem. Mater.  2021, 33, 5542-5554 |
| FeCo-NSC | – | 0.860 | Energy Stor. Mater.  2022, 45, 805-813 |

**Table S7.** Summary of some recently reported representative Zn-air batteries.

| **Cathode**  **catalysts** | **Peak power density**  **(mW cm^-2^)** | **Galvanostatic discharge-charge time**  **(h)@ 5 or10 mA cm^-2^** | **Reference** |
| --- | --- | --- | --- |
| **PtFe_NPs_@PtFe_SAs_-N-C** | **298** | **1000@5** | **this work** |
| Fe-N_4_/Pt-N_4_ | 200 | 40@10 | Angew. Chem. Int. Ed.  2021, 60, 19262-19271 |
| Pt-SCFP/C-12 | 122 | 80@5 | Adv. Energy Mater.  2020, 10, 1903271 |
| R_0.1_ZIS | 176 | 262@10 | Adv. Funct. Mater.  2022, 2110572 |
| Fe SAs/NPs@NC | 107.9 | 180@10 | Chem. Eng. J.  2023, 456, 140858 |
| PdMo bimetallene/C | 154.2 | 500@10 | Nature  2019, 574, 81-85 |
| FeSA/N-PSCS | 164.5 | 140@10 | Energy Stor. Mater.  2023, 59, 102790 |
| NDGs-800 | 115.2 | 78@10 | ACS Energy Lett.  2018, 3, 1183-1191 |
| Fe/N-G-SAC | 120 | 200@10 | Adv. Mater.  2020, 32, 2004900 |
| N-CoS_2_ YSSs | 81 | 160@10 | Adv. Sci.  2020, 7, 2001178 |
| Fe_3_C-FeSA@3DCN | 166 | 200@5 | ACS Appl. Mater. Interfaces  2023, 15, 5720-5731 |
| Fe,Co,N–C | 198.4 | 48@5 | Appl Catal. B  2019, 244, 150-158 |
| FeN_x_/C-700-20 | 36 | 84@5 | Adv. Energy Mater.  2018, 8, 1800955 |
| Fe–N–NDC-1-900 | 266 | 160@5 | J. Mater. Chem. A,  2021, 9, 5556-5565 |
| FeNi_3_@NC | 139 | 30@10 | Appl Catal. B  2020, 268, 118729 |
| FeP/Fe_2_O_3_@NPCA | 130 | 100@5 | Small  2023, 2300136 |
| Zn/Co–N@PCNFs-800 | 83.5 | 24@10 | [Nano-Micro Lett](https://link.springer.com/journal/40820).  2019, 11, 8 |
| Fe-N-C | 175 | 643@5 | Appl Catal. B  2022, 313, 121454 |

**5. References**

[1] P. Giannozzi, O. Andreussi, T. Brumme, O. Bunau, M. B. Nardelli, M. Calandra, R. Car, C. Cavazzoni, D. Ceresoli, M. Cococcioni, *J. Phys. Condens, Matter.* **2017**, *29*, 465901.

[2] P. Giannozzi, S. Baroni, N. Bonini, M. Calandra, R. Car, C. Cavazzoni, D. Ceresoli, G. L. Chiarotti, M. Cococcioni, I. Dabo, *J. Phys. Condens, Matter.* **2009**, *21*, 395502.

[3] J. Paier, R. Hirschl, M. Marsman, G. Kresse, *J. Chem. Phy.* **2005**, 122.

[4] J. P. Perdew, K. Burke, M. Ernzerhof, *Phys. Rev. Lett* **1996**, *77*, 3865.

[5] P. E. Blöchl, Phys. Rev., B Condens, *Matter.* **1994**, *50*, 17953.

[6] S. Grimme, J. Antony, S. Ehrlich, H. J. T. J. o. c. p. Krieg, *The Journal of chemical physics.* **2010**, *132*.

[7] S. Grimme, S. Ehrlich, L. Goerigk, *J. Comput. Chem.* **2011**, *32*, 1456.

[8] V. I. Anisimov, F. Aryasetiawan, A. I. Lichtenstein, *J. Phys. Condens, Matter.* **1997**, 9, 767.

[9] V. I. Anisimov, J. Zaanen, O. K. Andersen, *Phys. Rev., B Condens, Matter.* **1991**, *44*, 943.

[10] H. Hsu, K. Umemoto, P. Blaha, R. M. Wentzcovitch, *Earth Planet. Sci. Lett.* **2010**, *294*, 19.
